# Twists and Turns of Liquid Crystals Unravelled by Small‐Angle Scattering

**DOI:** 10.1002/smtd.202501808

**Published:** 2026-01-10

**Authors:** Jessie Wong, Jean‐Luc Brousseau, Hatem M. Titi

**Affiliations:** ^1^ Department of Chemistry McGill University Montreal Quebec Canada; ^2^ Anton Paar Ashland Virginia USA

**Keywords:** grazing incident, liquid crystals, material sciences, particles, self‐assembly, X‐ray scattering

## Abstract

X‐ray scattering is a highly versatile characterization method and has seen widespread use across all fields of science. Previous review articles pertaining to small‐ and wide‐angle X‐ray scattering (SWAXS) have either been highly specific or narrow in scope. Generally, other SWAXS reviews have been mainly tailored toward characterizing biological protein samples or polymers. However, there appears to be a literature gap in how SWAXS may be used in characterizing self‐assembled systems, more specifically, liquid crystals. SWAXS is a crucial technique used for characterizing liquid crystals, offering valuable crystallographic insights that cannot be directly observed by optical or spectroscopic methods. Unlike spectroscopic techniques, SWAXS can provide valuable nanoscale structural information over a larger volume of material, and it will be discussed in detail herein. This review seeks to fill that gap as well as aid in educating and welcoming prospective scientists interested in learning to use the technique for materials characterization. Several studies will be covered on how SWAXS was used to characterize the most common self‐assembled phases.

## Graphical abstract

X‐ray scattering provides valuable insights into material structure and self‐assembly. This review discusses the use of small‐ and wide‐angle X‐ray scattering (SWAXS) as well as grazing incident SWAXS for examining self‐assembled systems, especially liquid crystals. It demonstrates how SWAXS reveals structural organization across various scales, enabling scientists to utilize this versatile technique for advanced materials analysis.

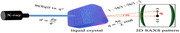

## 1 Introduction

Despite the increasing accessibility of more modern and advanced instrumentation and software, X‐ray scattering techniques, such as small‐ and wide‐angle X‐ray scattering (SAXS and WAXS), remain underutilized in the field of chemistry [1]. Chemistry education at the undergraduate level mandates commonly used characterization techniques such as nuclear magnetic resonance (NMR) and mass spectrometry  (MS) while neglecting uncommon techniques, largely spectroscopic and X‐ray‐based instrumentation. As a result, chemistry students often disregard materials chemistry for more “traditional” synthetic‐based fields. This review seeks to realize the power and significance of SAXS and WAXS in nanoscale materials characterization [2, 3, 4, 5, 6]. We will provide a surface‐level introduction to theory as well as a practical guide toward using these techniques in characterizing difficult material samples, focusing on liquid crystals. We want to emphasize that this review objectively examines both lab‐based and synchrotron techniques from an unbiased perspective of a specific manufacturer.

**FIGURE 1:**
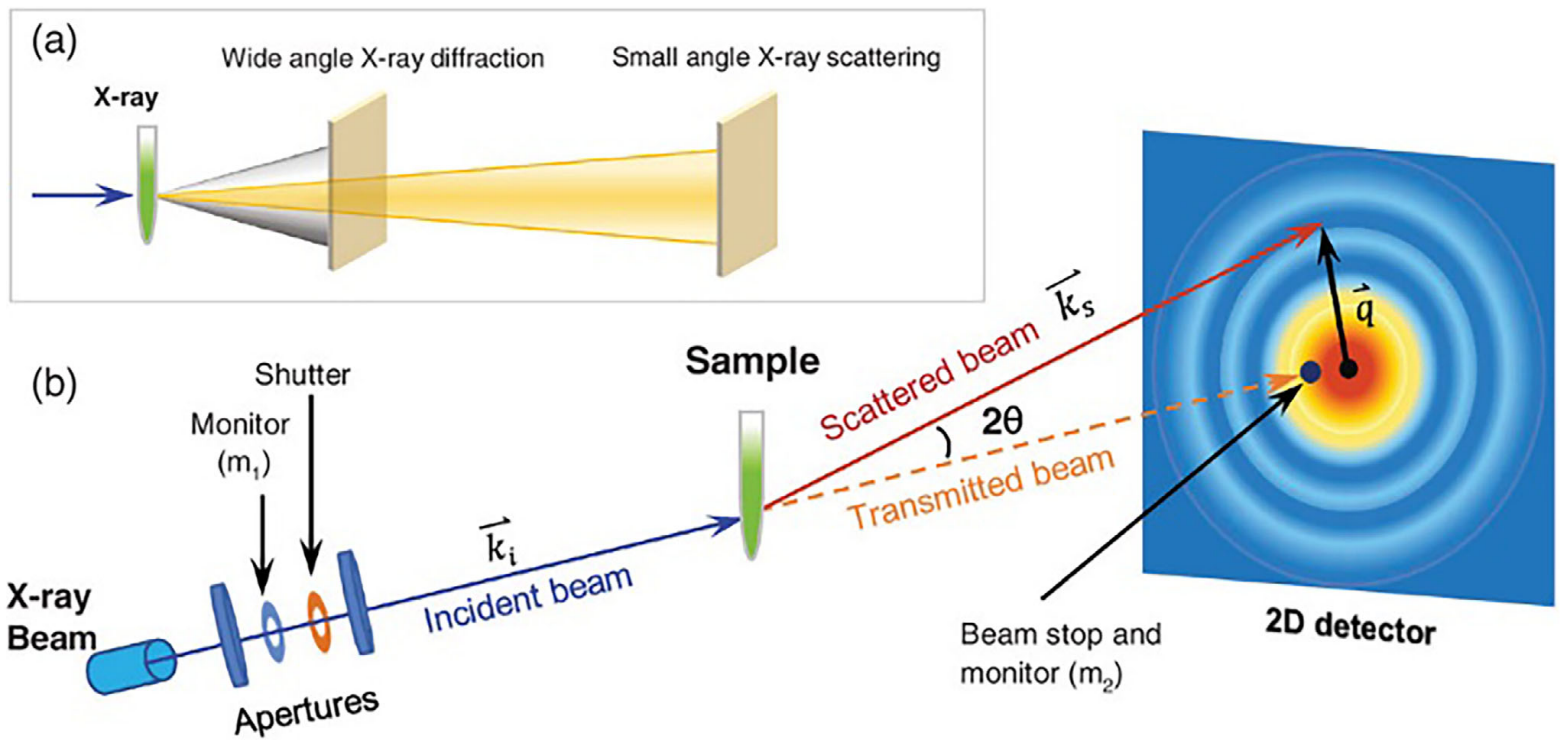
Experimental differences between small‐ and wide‐angle X‐ray scattering (SAXS and WAXS), in which the distance of the sample from the detector can dictate whether the experiment is running in SAXS or WAXS mode. (a) Schematic of the difference in sample‐to‐detector distances (SDD) in laboratory‐based SWAXS. (b) SAXS setup for a synchrotron beamline at the Advanced Photon Source. Reproduced with permission [7]. Copyright 2020, Wiley.

First and foremost, SAXS and WAXS (SWAXS for simplicity), as well as X‐ray diffraction (XRD), utilize elastic X‐ray scattering to probe different length‐scale features of a sample. Depending on the experimental setup, all these techniques could potentially be performed on one instrument (Figure 1) [5, 7]. For crystal structure and crystallinity of the materials, XRD would be the go‐to experimental technique, as a detector would collect all angles (generally, the angles in XRD are expressed in 2*θ* and range typically between 3° and 120°), thereby allowing for a high‐resolution two‐ and three‐dimensional electron density maps, which can eventually lead to a crystal structure solution. The drawback, however, is that all angles must be independently scanned, and this results in long acquisitions (up to several hours). Additionally, XRD is mostly limited to crystalline materials, so preparation of the sample is often restricted to high‐quality crystals.

Conversely, experimental times in SWAXS are short (>90 s; or a fraction of a second in case of synchrotrons) since only one snapshot is taken [8]. The difference between SAXS and WAXS is the sample‐to‐detector distance (SDD), since the scattering angle is inversely proportional to the length of the feature being studied (i.e. a smaller feature results in larger scattering angles) [9]. For SAXS, the sample is placed far from the detector such that the resulting X‐ray scattering profile covers small angles (typically <0.1° to 5°). This set‐up probes structural features from approximately one to hundreds of nanometers, which include intermolecular spacings, shapes and sizes, voids, and ordered assemblies [6]. However, it is important to note that there is no agreement on the exact transition values between SAXS and WAXS, neither in terms of *q* or d‐space. Conversely, WAXS is measured with the sample and the detector closer to each other, resulting in larger scattering angles of the incident X‐ray, which allows for probing of smaller features, such as atomic lattices, phase compositions, and crystallinity. Essentially, SWAXS is a probe of inhomogeneities in the electronic densities, which allows for characterization of a wide library of properties.

To understand how X‐ray scattering is connected to features probed by SWAXS, an understanding of Bragg's law is necessary. The Bragg condition occurs when successive atomic planes scatter X‐ray radiation such that the reflected X‐radiation interferes constructively and can be described by the following equation, Equation (1), where *n* is an integer describing the diffraction order (1, 2, 3 etc.), 𝜆 is the wavelength of the incident X‐ray radiation, and *θ* is the angle between the incident beam and the atomic plane. In a lab setting, 𝜆 represents a commonly used source of Cu*K*ɑ radiation and corresponds to 𝜆 = 1.5418 Å. From this relationship, we can see the characteristic powder pattern seen in X‐ray Diffraction (XRD) [5, 7, 10].

(1) nλ=2dsinθ

To probe structural features, Equation (1) may be rearranged such that the scattering vector, *q*, may be used to measure interacting distances through Equation (2). From here, the scattering vector can be related to real space through Equation (3) and represents the interacting distance [11, 12]. The relationship between Equations (2) and (3) explains why larger scattering angles are used to probe small distances and vice versa. For example, a real distance of 5000 nm would produce a scattering vector of 0.00126 nm^−1^, while 50 nm would produce a value of 0.126 nm^−1^ [13].

(2) $$q=\frac{4\pi }{\lambda }\sin\theta$$

(3) $$d=\frac{2\pi }{q}$$

**FIGURE 2:**
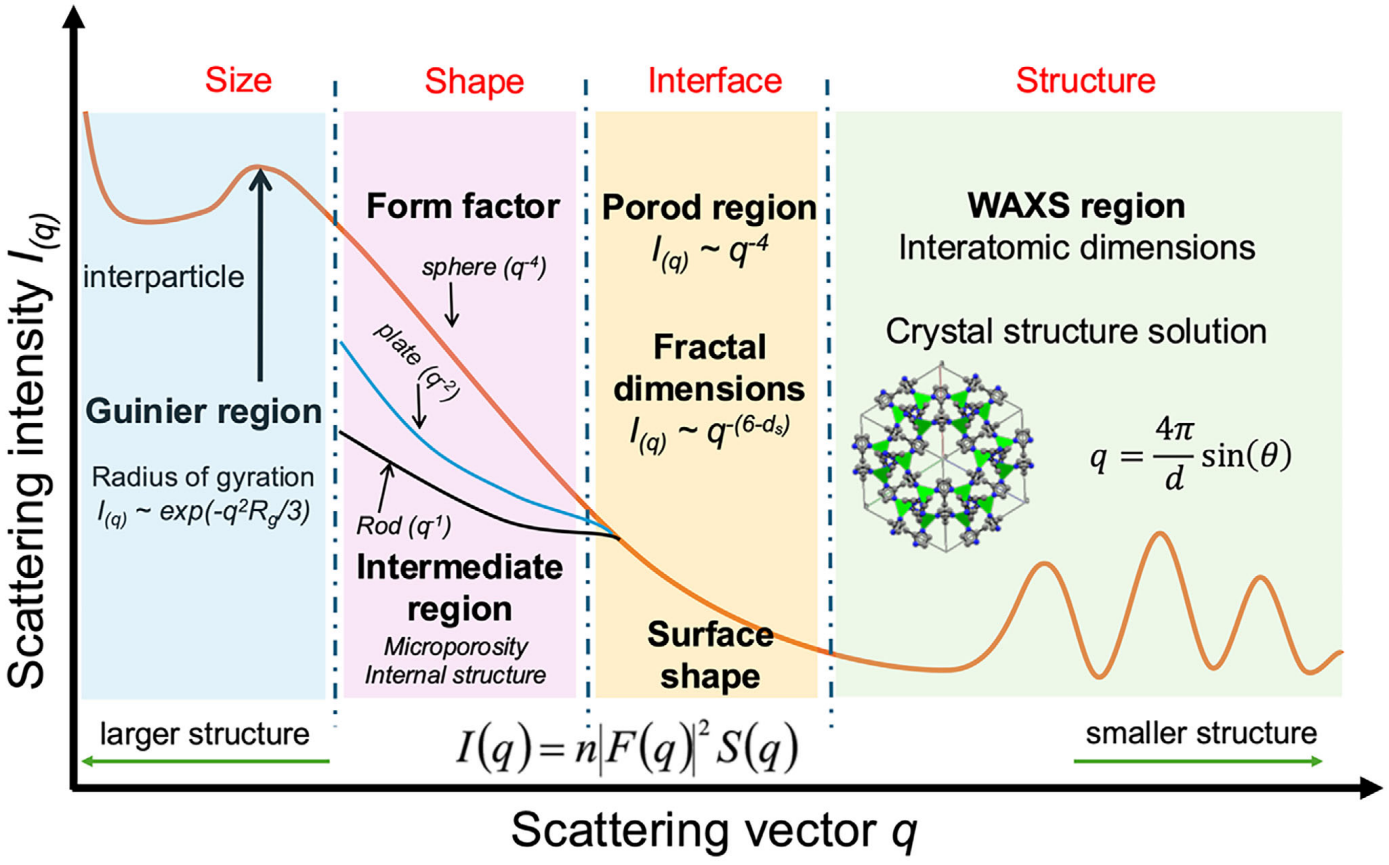
Schematic diagram depicting the useful fitting regions of a hypothetical small‐angle X‐ray scattering (SWAXS) profile spanning small and wide angles. Depicted here are the Guinier and Porod regions, where the threshold occurs at R_g_*q =1.3, as well as other useful regions that are useful for linear fitting [30]. It is important to note that the schematic is simply a visual aid; *q* values, order and presence of these regions vary with real experimental data.

While SWAXS machine collects a two‐dimensional map of scattered X‐ray radiation, typically the scattering profile is reduced to a one‐dimensional plot as seen in Figure 2. The advantage of this data reduction is that the three SAXS regions become available for study and measurements made at different wavelengths are directly comparable. In order of increasing *q* values, these are the Guinier [14], Intermediate [15, 16], and Porod regions [17, 18], which allow for the determination of the size (via the radius of gyration), shape (through pair‐distance distribution functions) and interfaces/surfaces (by analyzing the intensity of scattered X‐rays in this region), respectively. It is also important to note that the ranges of *q*‐values within each region are relative and depend on the sample being studied. Resources on the fundamentals of these regions and their respective relationships are numerous, and we direct the reader to the following references [19, 20, 21, 22].

It is also sometimes useful to distinguish the scattered X‐rays as arising from the form factor or the structure factor [20]. Scattering resulting from particle shape, size, etc., is the form factor. Conversely, scattering arising from periodic lattices (either from atomic unit cells or ordered nanostructures) comprises the structure factor. If the form factor is of interest, the sample is prepared in a dilute solution to minimize the structure factor's contributions; this is especially important in characterizing protein structures and other ordered assemblies [13, 23, 24, 25, 26, 27]. Similarly, for materials characterization, the form and structure factors may overlap, and so the molecular geometry/dimensions of the sample in question is determined beforehand as to exclude scattering arising from shape/size [26, 28, 29].

Since the topic of this review is SAXS, it is worthwhile to mention the major benefits and drawbacks of using SAXS compared to other characterization methods. Firstly, the length scale that can be probed with SAXS spans up to hundreds of nanometers (or up to the micron range using Ultra SAXS (USAXS)) [10, 31], which allows for a thorough investigation of the entire structure and all the relevant parameters. Secondly, the technique can nondestructively examine a variety of parameters within a high‐resolution spectrum. Intermolecular distances [32], porosity [33], conformations [34], size and shape [35, 36], and agglomeration [37] measurements become possible, just to name a few. Alongside other characterization methods, SAXS can be used to provide a more complete picture of the sample in question. While SAXS can investigate a wide and vast array of properties, the instrument is not perfect. SAXS is not accessible to all researchers, as not all labs are able to afford a laboratory SAXS instrument. As a result, the sample may need to be sent to a commercial characterization lab or even a beamline, in which case scheduling and pricing may prohibit use. In addition to the economic inaccessibility, SAXS data are also generally difficult to interpret. Since all scattered radiation is collected at once, it could potentially be difficult to decipher what feature is responsible for the scattered peaks and this drawback is exacerbated for multiphase systems. In terms of the 1D data reduction, there are also several relationships that can be used at different *q*‐values to extract various measurements. To summarize, Pauw states: “Of the three most‐wanted morphological aspects: (1) shape, (2) polydispersity, (3) packing, two must be known or assumed to obtain information on the third” [10]. Liquid crystals (LCs) are particularly difficult samples to characterize using SAXS for this reason, and there is yet a resource that compiles all the knowledge for this purpose.

### 1.1 Liquid Crystals (LCs)

Liquid crystals (LCs) are vital in 21^st^ century technologies. Since the incorporation of LCs into display technologies in the 1980s (replacing cathode ray tubes), LC displays have become dominant in our personal electronic devices such as computers, smartphones, tablets, etc. While other display technologies (namely QLEDs and OLEDs) have been developed to replace LC displays, LCs have found uses in other fields due to their electro‐optical/stimuli‐responsive properties. For example, flexible displays, smart windows, LC lenses, biosensors, and “4D‐printing” have found ways to incorporate LC materials [38, 39, 40, 41].

**FIGURE 3:**
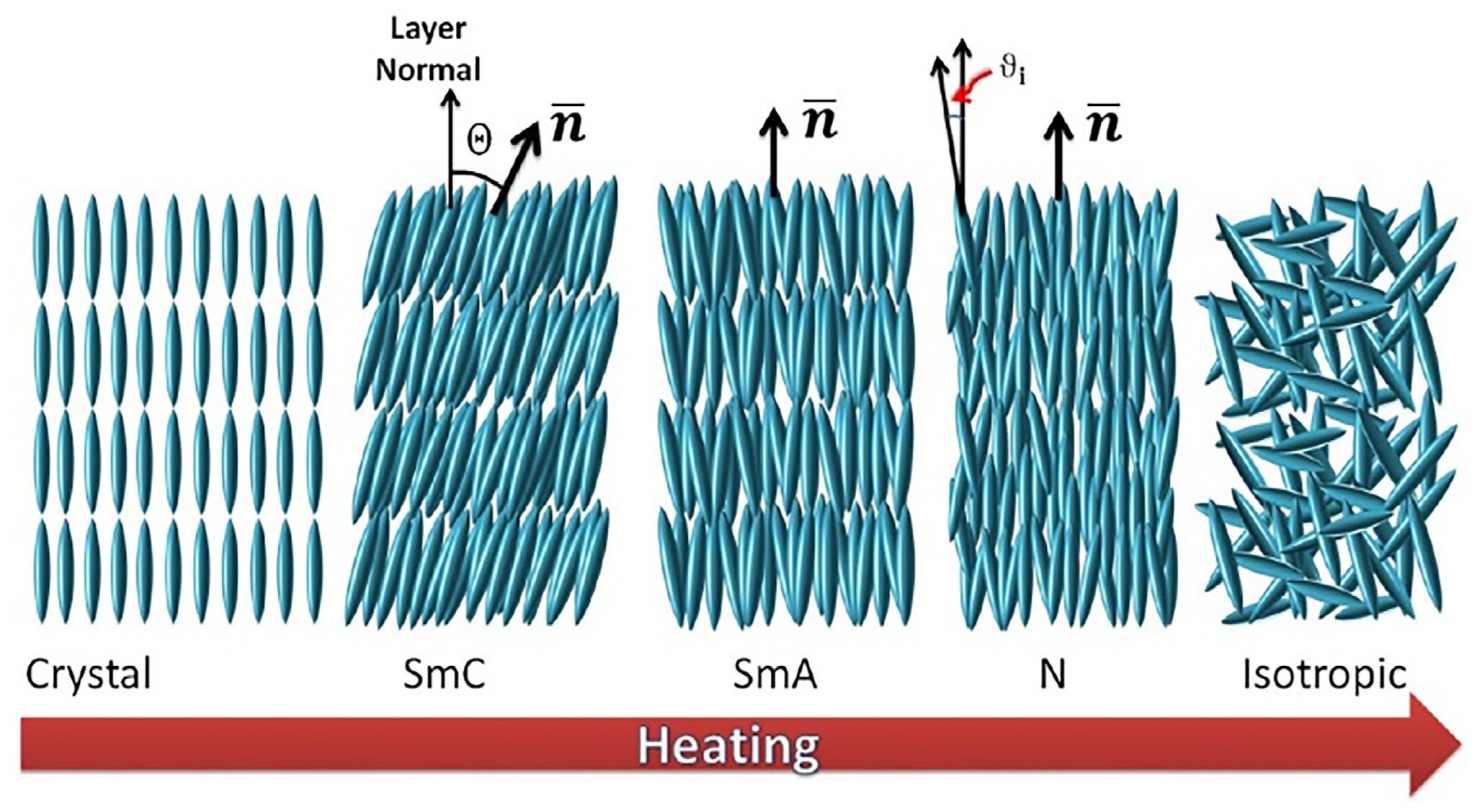
Thermotropic liquid crystals (LC) that undergoes several phase transitions upon heating. This schematic representation illustrates the transition from a 3D crystal lattice with fixed molecular positions to 1D lattices (layers) where the molecules are assumed to be rotational in addition to some translational mobility, known as smectic, before this positional order is lost to form a Nematic (N) lattice which retains only long‐range orientational order. Further heating leads to isotropic, disorderly‐type structures. These structures exhibit unique scattering patterns, which may be monitored using X‐ray techniques. Note that Smectic phases are SmC and SmA, while the nematic phase is N. Reproduced under the terms of the CC‐BY 4.0 license [52].

The reason why LCs are popular nanocomposite materials is due to their structural properties. LCs are essentially ordered fluids, where the molecule simultaneously has the structure/rigidity to form ordered assemblies, while also having the mobility to self‐assemble into the ordered structure. LCs may be distinguished as either thermotropic or lyotropic, in which their self‐assembly behavior is attributed to temperature changes, or upon reaching a sufficiently high concentration in solution, respectively. The molecular structure of an LC will determine whether the behavior will be thermotropic or lyotropic. For example, rigid molecules with anisotropic cores and relatively low melting points, such as 5CB (4‐cyano‐4'‐pentylbiphenyl), will form thermotropic LCs [42], while rigid molecules that are soluble within a chosen solvent will form lyotropic LCs, such as cyclodextrin [43] or cellulose [44, 45]. Furthermore, lyotropic LCs also include self‐assembled structures comprised of amphiphilic surfactants [46, 47]. Of the two LC behaviors, LCs can be further classified into ordered structures (shown in Figure 3). For example, rod‐shaped molecules can form nematic LCs, where the molecules are oriented, but with no periodic structure, and the nematic LC may be either thermotropic or lyotropic. For other, less common LC structures, of the available LC phases, each LC may be characterized through a combination of intermolecular distances, order parameters and chirality, etc. We aim to guide the reader to several comprehensive overviews of LC materials and their phase structures, which are well documented in the literature [48, 49, 50]. Notably, the section “Structural Studies of Liquid Crystals by X‐ray Diffraction” in “Handbook of Liquid Crystals” provides a detailed explanation of different liquid crystal phases and their X‐ray diffraction patterns [51].

Polarized optical microscopy (POM), a technique in which the birefringence of LC materials produces unique optical textures under cross‐polarizers, is frequently used to identify and distinguish these textures. However, this technique requires a highly trained eye and a strong understanding of how these textures arise. For a comprehensive review of LC optical textures, we recommend the work of Dierking [53]. Various nuclear magnetic resonance (NMR) experiments are also commonly used to probe the orientation, structure, and dynamics of the LC in question [54, 55, 56, 57]. Additionally, more specific instruments/techniques may be selected for some unique LC systems, for example: neutron scattering [58], Mossbauer spectroscopy [59], dielectric measurements [60], and rheology [61], among many others. Many methods exist for characterizing LCs. Yet, there is no universal technique that works for all phases of LCs and there are drawbacks that come with each characterization method. For example, “traditional” NMR characterization is limited to only non‐chiral LCs, since the magnetic fields used in NMR will unwind cholesteric LCs and convert these LCs into nematic phases instead, phase N [62, 63]. Similarly, while Mossbauer spectroscopy can determine the mobility and orientation distribution of LCs, this technique is limited only to certain nuclei, so by design, LCs must incorporate the relevant nuclei [59, 64].

In the context of LC materials, SAXS is versatile in that the method is compatible with many different types of samples, and several LC properties can be extracted from the scattering profile. This review seeks to cover the various LC properties of LCs and LC nanocomposites and how they can be characterized using SAXS. Herein, we present simple LC systems as examples and describe how SAXS was used to characterize them. The selected examples are drawn from classical and historical systems in the literature and also from the past 5 years.

## 2 SWAXS Analyses and Fundamentals

The biggest challenge toward using SAXS in materials characterization is data interpretation [5, 10, 24], and for this reason, a bottom‐up approach will be used to cover all the facets of SAXS characterization. Firstly, it is important to understand how the scattered radiation appears and how it is represented. Within each measurement, the resulting data arises from scattering, which occurs from everything encountered by the path of the beam, which includes the sample, the solvent, and the sample holder itself (i.e. the walls of a capillary, etc.) [8]. For this reason, background correction becomes important, as to subtract the scattering contributions from the solvent and the sample holder. Toward data correction, Pauw provides a wonderfully succinct data reduction/correction process [10]. Additionally, it is important to note that any observed signal arises due to electron density contrast/difference. For biological/solution scattering, the electron density contrast arises from the difference in electron densities between the solvent and the sample. In the case of materials/bulk scattering, the difference arises from the material and vacuum/air which has a much stronger contrast, allowing for faster measurements. Periodic features within a bulk sample are amplified by constructive interference, while signals arising from the rest of the sample experience destructive interference, resulting in no observable signal in these regions [23].

### 2.1 Solution Scattering

Following data correction, interpretation naturally follows. One of the main challenges in experimental SAXS is the absence of a universal approach to data interpretation. This is largely because of SAXS's versatility, as the wide variety of materials and samples makes broad generalizations ineffective. For this reason, interpretation can be primarily distinguished between data arising from solution scattering or bulk/materials scattering. Firstly, solution‐based scattering generally applies to structural biology samples, and experiments are run in dilute solutions (typically 0.1–10 mg/mL) [65, 66]. Interpretation of solution scattering data is not immediately obvious; fitting of the data is frequently required. Prior to the availability of high‐performance computers, data interpretation was achieved through linear fitting of different regions of the 1D scattering profile. This is the origin of the “Porod”, “Guinier” and “Kratky” plots [10]. Fitting of this type requires that the data is replotted such that a linear relationship can be established. A summary of common linear fits is provided in Table 1. The examples mentioned are commonly encountered in many SAXS guides [66, 67], and are not an exhaustive list.

**TABLE 1:** List of common linear plots used in solution SAXS.

Analysis	Plot	Relationship	Information Provided	Refs.
Guinier	ln(I(q)) vs. *q* ^2^	$I\left(q\right)\approx I\left(0\right)e^{-\frac{q^{2}R_{g}^{2}}{3}}$ (for low *q* values; *R*_g_*q < 1.3)	Radius of Gyration, *R*_g_, which can also be used to determine molecular weight.	[24, 68, 69]
Porod	log(*I*(*q*)) vs. log(*q*)	*I*(*q*) ∝ *q^−^* ^α^	Surface or mass fractals, as well as polymer conformation	[70, 71]
Kratky	*q* ^2^ *I*(*q*) vs. *q*	$Q=\int_{0}^{\infty }q^{2}I\left(q\right)dq$	Globularity/flexibility determined from shape of curve. Area under curve provides Porod invariant, which can then be used to calculate the volume or surface‐to‐volume ratio of the scattering particle and finally molecular weight	[8, 72]
Porod–Debye	*q* ^4^ *I*(*q*) vs. *q* ^4^	$I\left(q\right)\approx \Delta \rho^{2}\frac{2\pi }{q^{4}}S$	Macromolecular volume can be determined by the plateau of the resulting plot.	[72]
Pair‐Distance Distribution	Fourier transform of *I*(*q*)	$G\left(r\right)=\frac{2}{\pi }\int_{0}^{\infty }Q\left[S(Q)-1\right]\sin\left(Qr\right)dQ$	Appearance of the resulting plot enables prediction of molecular size and shape	[15, 18]

Δ*ρ* = Electron density contrast; *S* = Surface area of the scattering particle; *α* = Fractal dimension exponent

### 2.2 Guinier Plot

Guinier's approximation establishes the scattering in the low *q* region (Guinier's region) as Equation (4).

(4) $$I\left(q\right)\approx I\left(0\right)e^{-\frac{q^{2}R_{g}^{2}}{3}}$$

By replotting the scattering data as ln (*I*(*q*)) vs. *q* ^2^, the radius of gyration (*R_g_*) can be obtained from the slope of the graph [68, 69]. It should be essential to note that the approximation only holds for when *R*_g_*q < 1.3 [20], which is determined iteratively. Noteworthy, the distinction between low vs. high q values is quite arbitrary and is determined by the sample of interest. Practically, a Guinier analysis is often limited to small sizes (<20 nm), as it is difficult to measure enough data at small *q*, for samples with large *R*_g_ values. It is very important to note that, for a sample with *R*_g_ = 100 nm, a Guinier plot would require enough measurements below a *q* value of 0.013 nm^−1^.

For monodisperse systems, where some information about the particle shape is known (from other characterization methods), *R*_g_ can be approximated if the dimensions of the nonspherical particles are known. For example, in the case of homogenous and monodisperse rods, the approximate *R*_g_ can be determined if the cross‐sectional radius, *R*, and length, *L*, of the rod are known through the following relationship: *R*_g_^2^ = *L* ^2^/12 + *R* ^2^/2. A list of geometries and their corresponding *R*_g_ relationships are listed in Table 2.

**TABLE 2:** Selected geometries and their corresponding radius of gyration expression.

Shape	*R*_g_^2^ expression
Rod (*L*, *R*) [73]	*L* ^2^/12 + *R* ^2^/2
Platelet (*t*, *L*) [73]	*L* ^2^/6 + *t* ^2^/12
Sphere (*R*) [74]	(3/5)*R* ^2^
Ellipsoid (*a*, *b*, *c*) [74, 75]	(*a* ^2^ + *b* ^2^ + *c* ^2^)/5
Hollow sphere (*R*_o_, *R*_i_) [74, 75]	(3/5)(R_o_^5^ ‐ R_i_^5^)/(R_o_^3^ ‐ R_i_^3^)
Hollow cylinder (*R*_o_, *R*_i_, *L*) [75, 76]	(*R*_o_^2^ + *R*_i_^2^)/2 + *L* ^2^/12
Elliptic cylinder (*a*, *b*, *c*) [75]	*a* ^2^/4 + *b* ^2^/4 + *c* ^2^/3

*L* = length; *R*, *R*_i_, *R*_o_ = radius, inner radius, and outer radius, respectively; *t* = thickness; *a*, *b*, *c* = semiaxes

### 2.3 Porod Plot

The high *q* linear portion of the scattering plot constitutes the Porod region. This region is governed by Equation (5), and by replotting the data as log(*I*(*q*)) vs. log(*q*), the fractal dimension, *α*, can be obtained by fitting the linear downward slope.

(5) $$I\left(q\right)=I\left(0\right)q^{-\alpha }$$

Determination of *α* can aid in structure determination, as *α* represents the behavior of the scattering system and whether this system follows a mass, surface, or pore fractal. For a system comprised of a large number of scatterers, scattering behavior is best understood by employing fractal theory and the fractal dimension, *α*, can then be qualitatively understood [70]. The physical interpretation of *α* is “a quantitative characterization parameter of the irregularity of complex forms used to indicate the validity of the space occupied by complex forms” [77]. However, a more practical definition only considers the non‐integer value of α and indexing the result to Table 3 [78, 79]. The fractal dimension, *α*, can be distinguished as mass, surface, and pore fractals and typically adopts a value between 0 < *α* < 4. Samples exhibiting 3 < *α* < 4 exhibit surface fractal behavior, while 0 < *α* < 3 can be either be considered pore or mass fractals (which can be disambiguated with more information). Broadly, in the lower limit of mass fractals where *α* = 0, scattering is largely dominated by discrete scatterers which are relatively far apart from each other while the upper limit (*α* = 3) indicates denser and more compact scatters. Values near *α* = 1, and *α* = 2, indicate stringy and disk‐like behavior, respectively [79, 80]. Similar analysis can be applied to pore fractals as well [77]. In contrast, values between 3 < *α* < 4 constitute surface fractals and can be a gauge for surface roughness, with a smooth surface occurring at *α* = 4 and a rough surface occurring at *α* = 3 [81].

**TABLE 3:** Summary of interpretations of fractal dimension exponent, *α*.

Porod exponent, *α*	Meaning
4	Mass with smooth surface
3	Mass with rough surface
3	Compact
2	Flat
1	Stringy

### 2.4 Kratky Plot

A Kratky plot may give information about the sample's globularity/flexibility based on the shape of the *q* ^2^ *I*(*q*) vs. *q* plot. A flexible unfolded polymer/protein will appear roughly linear, whereas a globular protein/polymer will give a distinctive peak shape [80]. Furthermore, integration of the curve yields the Porod Invariant, *Q* (Equation 6), which can then be used to calculate the volume and the surface‐to‐volume ratio of the scattering particle through the following equations.

(6) $$Q=\int_{0}^{\infty }q^{2}I\left(q\right)dq$$

One application of the invariant (and the Kratky plot) is molecular weight (SAXSMoW) determination of monodisperse proteins in dilute solution through Equation (7) [82]. Where *I*(0) is the zero intensity, *Q* is the invariant, and *ρ*_m_ is the average protein density (1.37 g/cm^3^). One advantage of this molecular weight determination method is that it is not necessary to know the concentration of the solution, however, the solution must be dilute enough that there are no potential structure factor contributions. Additionally, SAXSMoW is a simple and highly useful online tool for protein molecular weight determination [83, 84].

(7) $$Mw=2\pi^{2}\frac{I\left(0\right)}{Q}\rho_{m}$$

Alternatively, a relatively new and simple method of determining molecular weight for polydisperse non‐protein samples has recently been reported [85] This method does not require the Porod invariant, but it does require that the resulting 1D plot be normalized to its known concentration. This method is outlined in Equation (8). To put simply, the numerator is the zero‐intensity determined for the fitted curve, whereas the denominator is the difference of the area under the curves for the experimental and fitted data in the concentration‐dependent regime. This concentration‐dependent region is defined in Equation (8) by *q*_m_, which the authors determined by calculating the cumulative invariants between the experimental and fitted data. The largest drawback of this method is the sensitivity of the low *q* region to polydispersity and aggregation.

(8) $$Mw=N_{A}\frac{I_{\mathrm{fit}}\left(0\right)}{\frac{1}{2\pi^{2}}\left(\int_{0}^{q_{m}}\frac{1}{c}I_{fit}\left(q\right)q^{2}dq-\int_{0}^{q_{m}}\frac{1}{c}I_{exp}\left(q\right)q^{2}dq\right)\;}$$

With the increasing availability of high‐performance computers, more intricate analyses may be carried out with relative ease. This includes modeling and sophisticated data fitting, extending beyond basic linear relationships, using a variety of available software tools [86]. It is important to note that molecular fitting and simulations suffer from various challenges, including overfitting, overreliance on forward models, and unknown confidence intervals [87].

### 2.5 Pair‐Distance Distribution Function (PDDF)

Toward understanding the size and shape of a material, a pair‐distance distribution function (PDDF) may be obtained. In order to describe the PDDF, it is important to define the radial distribution function, R(r), and it can be thought of as a “histogram of every interatomic distance in a material” [18]. In other words, every atomic distance pair in a material is “counted” and represented as a histogram. Alternatively, a normalized R(r) can be obtained by only considering the atoms within a sphere with a radius, *r*, and this is the PDDF, G(r) (or sometimes P(r)), see Equation (9).

(9) $$G\left(r\right)=\frac{R\left(r\right)}{r}-4\pi r\rho_{0}$$

Practically, G(r) is obtained from an indirect sine Fourier transform relationship (Equation 10), where S(Q) is the total scattering structure function, and Q is the scattering vector (Equation 2) [18, 88].

(10) $$G\left(r\right)=\frac{2}{\pi }\int_{0}^{\infty }Q\left[S\left(Q\right)-1\right]\sin\left(Qr\right)dQ$$

**FIGURE 4:**
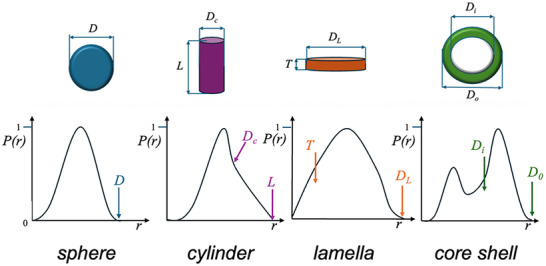
Pair‐distance distribution profiles of selected geometries. For each of these selected geometries, specific distances and sizes can be easily determined for homogeneous and monodisperse samples. The parameters *D*, *L*, and *T* refer to the shape's diameter, length, and thickness, respectively.

In short, the PDDF is easily achievable from the obtained SAXS data, which in turn enables the determination of size and shape measurements through the shape of the resulting PDDF profile [13]. A schematic of various geometries and their expected PDDFs is depicted in Figure 4.

### 2.6 Bulk/Materials Scattering

**FIGURE 5:**
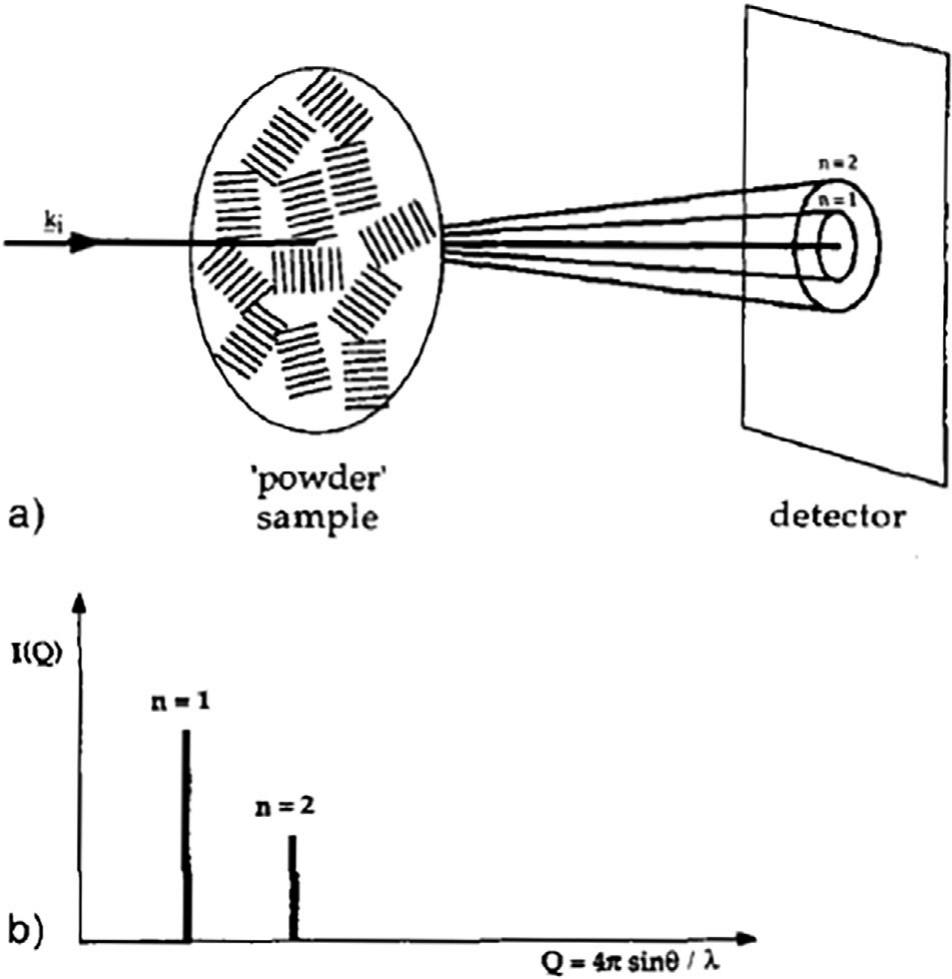
(a) Schematic representation of Bragg diffraction arising from an unaligned, polydomain crystalline sample. The concentric 2D scattering profile emerges due to a superposition of all the orientations of the crystalline domains within the sample. (b) Resulting 1D scattering profile from radial integration of the sample. Reproduced with permission [49]. Copyright 1998, Wiley.

Within ordered nanostructures, there are several features which satisfy Bragg diffraction, and each of these features may result in a signal. The scattering profile can primarily be represented in two ways, the 2D pattern, which is directly obtained from the detector (Figure 5a), or alternatively, it may be useful to perform a data reduction to convert the results to a 1D profile (Figure 5b). Commonly, the 2D pattern can be used to visualize alignment/orientation or periodicity of the LCs, but it is difficult to correlate the features with a measurable length.

**FIGURE 6:**
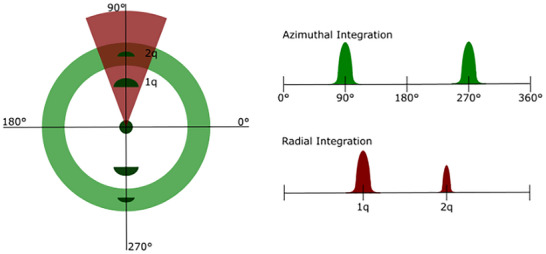
Schematic diagram of azimuthal (green) and radial (red) integration (Left) and the resulting 1D scattering profile (Right).

A 1D scattering profile, instead provides either orientational information via azimuthal integration, or aids in structural identification/feature measurements through radial integration. Both types of integrations involve data reduction, focusing only on the relevant sections to optimize data resolution (Figure 6) [89, 90]. Azimuthal integration involves the conversion of data in Cartesian space to polar coordinates, in other words, the summation of the scattered intensities concentric about the transmitted beam. Data reduction following radial integration translates the *x* and *y* lengths of the detector pattern to its scattering vector, *q* (see Equation 2). This produces a 1D scattering profile where the intensity is plotted against the scattering vector, *q*, where each peak represents a Bragg reflection from a structural feature. The peaks can then directly be translated to a real length scale, *d*, through the relation in *d* = 2π/*q* [10]. In contrast, azimuthal integration (Figure 6) of the scattered intensities provides a distribution of LC preferential orientations (full width at half maximum, FWHM, is used to calculate the crystallite orientation). Furthermore, the order parameters of the LC system are related to the azimuthal integration via Herman's algorithm (Equation 11) [91, 92].

(11) $$f_{a}=\frac{\int_{0}^{\pi }\frac{3}{2}\cos^{2}\varphi -\frac{1}{2}\sin\varphi I\left(\varphi \right)d\varphi }{\int_{0}^{\pi }\sin\varphi I\left(\varphi \right)d\varphi }$$

The line shapes obtained from radial integration reveal translational order within the system. In a perfect crystal, highly periodic and intense peaks of many diffraction orders are observed, however peaks observed in experimental 1D profiles tend to skew toward lower *q*, due to molecular motion [49]. This long‐range order is typically seen in smectic phases and amphiphilic LCs owing to the periodic structures, though higher order peaks may not always be observed in these systems. Conversely, short‐range order is frequently observed in LCs as broad peaks. Correlation lengths describe the length of positional ordering and can be estimated from the signal width at half‐maximum (FWHM, Δ*q*) of the peaks using the Scherrer equation or from *L* = 2π/Δ*q* [93].

### 2.7 Crystallography Basics

In contrast to XRD, which measures the scattered intensity in 2*θ*, SAXS instead uses the scattering vector, *q*. Furthermore, characteristic peak ratios are used to identify nanostructures in SAXS, in place of crystallographic reflections. Toward understanding the origin of the peak ratios, cubic space groups provide the most straightforward examples. The Miller indices of cubic systems can be described as:

(12) $$q_{\left(\mathrm{hkl}\right)}=\frac{2\pi }{a}\;\sqrt{h^{2}+k^{2}+l^{2}}$$

**SCHEME 1:**
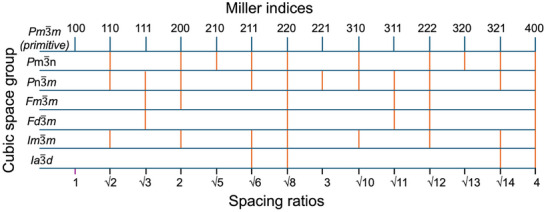
Selected cubic phases in SAXS, their allowed Bragg reflections in comparison to the primitive cubic system, and corresponding spacing ratios.

If we want to determine the peak ratios for the space group *Pn*‐3*m*, we need to determine the observed reflections (and absences). The observed reflections for the cubic space groups are depicted in Scheme 1. As can be seen, the first three observed reflections of $Pn\bar{3}m$ occur at Miller indices (110), (111), and (200), and since the primary reflection occurs at (110), q* becomes q*_(110)_. It is important to note that peak ratios in the literature do not always distinguish between normalized and non‐normalized peak ratios, so it is important that the reader(s) understand how peak ratios are calculated. The normalized peak ratios are normalized to the primary peak and are determined via for example *q*_(111)_/*q*_(110)_, *q*_(200)_/*q*_(110)_, whereas the non‐normalized peak ratio includes the primary peak, *q*_(110)_, and the peak ratio follows as *q*_(110)_, *q*_(111)_, *q*_(200)_. The general expression to determine peak ratios in *Pn*‐3*m* is depicted in Equation (13) and the first three peak ratios are calculated via Equations ((14), (15), (16)).

(13) $$\frac{q}{q*}=\frac{q_{\left(\mathrm{hkl}\right)}}{q_{\left(110\right)}}=\frac{\sqrt{h^{2}+k^{2}+l^{2}}}{\sqrt{1^{2}+1^{2}+0^{2}}}=\frac{\sqrt{h^{2}+k^{2}+l^{2}}}{\sqrt{2}}$$

(14) $$\frac{q_{\left(110\right)}}{q_{\left(110\right)}}=\frac{\sqrt{1^{2}+1^{2}+0^{2}}}{\sqrt{1^{2}+1^{2}+0^{2}}}=\frac{\sqrt{2}}{\sqrt{2}}=1$$

(15) $$\frac{q_{\left(111\right)}}{q_{\left(110\right)}}=\frac{\sqrt{1^{2}+1^{2}+1^{2}}}{\sqrt{1^{2}+1^{2}+0^{2}}}=\frac{\sqrt{3}}{\sqrt{2}}=\sqrt{\frac{3}{2}\;}$$

(16) $$\frac{q_{\left(200\right)}}{q_{\left(110\right)}}=\frac{\sqrt{2^{2}+0^{2}+0^{2}}}{\sqrt{1^{2}+1^{2}+0^{2}}}=\frac{\sqrt{4}}{\sqrt{2}}=\sqrt{2}\;$$

Consequently, in the case of $Pn\bar{3}m$, the normalization factor is defined relative to (110) and equal to √2, and we will see two types of conventions of (*q*/q*), where the first contains a normalized factor, while the other is absolute/non‐normalized, using the expression containing *hkl*, i.e., In Table 4, we have included both conventions for clarity. More complex examples are excluded, as they are outside the scope of the review. However, one should note that lower crystallographic systems, such as hexagonal systems, also depend on lattice parameters, not just on their Miller indices. Characteristic peak ratios are commonly used in identifying self‐assembled nanostructures in amphiphiles such as surfactants and copolymers, which will be discussed later. For other, more exotic structures, we direct the reader to the following references [94, 95, 96, 97, 98, 99]. Identifying the peak ratio of various columnar arranged phases is also very important, especially for discotic LC; therefore, we included the plane groups and their corresponding Bragg reflections (*q* *_hk_*) in Table 5 [100, 101, 102].

**TABLE 4:** List of selected supramolecular structures and their corresponding peak ratios observed in small‐angle X‐ray scattering (SAXS).

Structure	Structure type	Characteristic peak ratio non‐normalized (*q*/q*)	Characteristic peak ratio normalized (*q*/q*)
Lamellar [100]	Zero curvature	1 : 2 : 3 : 4	1 : 2 : 3 : 4 (same)
Hexagonal and inverse hexagonal (H_II_) (*p*6*mm*) [100]	Discontinuous	1 : √3 : √4 : √7 : √9	1 : √3 : √4 : √7 : √9
BCC (Body‐centered cubic, *Im* $\bar{3}$ *m*) [101]		√2 : √4 : √6 : √8	1, √2, √3, 2
FCC (Face‐centered cubic, *Fm* $\bar{3}$ *m*) [101]		√3 : √4 : √8 : √11	1, √(4/3), √(8/3), √(11/3)
Micellar cubic (*Fd* $\bar{3}$ *m*)		√3 : √8 : √11 : √12	1, √(8/3), √(11/3), √(12/3)
Disordered micellar [107]		Broad peak, no distinct ratios	
Gyroid phase (*Ia* $\bar{3}$ *d*) [101]	Bicontinuous cubic	√6 : √8 : √14 : √16	1, √(4/3), √(7/3), √(8/3)
Diamond phase (*Pn* $\bar{3}$ *m*) [101]		√2 : √3 : √4 : √6	1, √(3/2), √2, √3
Primitive phase (*Im* $\bar{3}$ *m*) [100]		√2 : √4 : √6 : √8	1, √2, √3, 2

*Note* that the p6mm is a plane group which is found in two‐dimensional periodic patterns, and there are seventeen unique plane symmetry groups, also known as wallpaper groups, full list is in Table 5.

**TABLE 5:** List of selected supramolecular structures of plane groups and their corresponding peak ratios observed in small‐angle X‐ray scattering (SAXS).

Lattice type	Plane group	q*_hk_* relation	Peak ratios (*q*/q*)	Miller indices
Oblique	*p*1, *p*2	√[*h^2^a* ^2^ + 2*hk ab cos*γ + *k^2^b* ^2^]	Values are depends on the parameters *a, b*, a *γ* and in the unit cell	All (*h*, *k*)
Rectangular (primitive)	*pm*, *pmm*, *pmg*, *pgg*, *pg*	√[(*h*/*a*)^2^ + (*k*/*b*)^2^]	(values are depends on the parameters *a* and *b* in the unit cell)	All (*h*, *k*)
Rectangular (centered)	*cmm*, *cm*	√[(*h*/*a*)^2^ + (*k*/*b*)^2^] where *h* + *k* = 2n	Depends on the parameters *a* and *b* in the unit cell	(1,1), (2,0), (0,2), (2,2), (3,1)
Square	*p*4, *p*4*mm*, *p*4*g*	√[h^2^ + k^2^]	1 : √2 : 2 : √5 : √8:3	(1,0), (1,1), (2,0), (2,1), (2,2), (3,0)
Hexagonal	*p*6, *p*6mm, *p*3, *p*3*m*1, *p*31*m*	√[*h* ^2^ + *hk* + *k* ^2^]	1 : √3 : 2 : √7 : √12 : 3	(1,0), (1,1), (2,0), (2,1), (2,2), (3,0)

This table provides a general overview of the plane groups and their corresponding peak ratios, without specifying how to distinguish between plane groups of the same family.

### 2.8 Software

The data, whether from a lab SAXS instrument or a synchrotron, need to be reduced from a 2D detector image to a 1D curve. Synchrotrons usually have their data‐reduction software in‐house, and commercial instruments also include data‐reduction programs with the package. As for data analysis, a commercial SAXS instrument offers basic data analysis. Synchrotron groups have developed different analysis tools for different types of material.

For SAXS of biological samples, the most common package is ATSAS [103], which delivers *P(r)*, ab initio shape, rigid‐body modeling, and more. The BioXTAS RAW software [104] is also excellent for Guinier analysis and SEC‐SAXS. There are general materials, small angle scattering programs like SasView [105] for form and structure factors, polydispersity, and the Irena software running on Igor Pro [106], which is a comprehensive SAS analysis software with great online tutorials. Other packages worth mentioning: FIT2D [107], SASfit [108], and GSAS‐II [109].

### 2.9 Advanced SWAXS Methods and Data Analysis Techniques

This section has highlighted several ways in which SWAXS may aid in characterization; however, several advanced characterization methods and analyses may also be undertaken (primarily in synchrotron‐based sources). For the sake of brevity, only a few examples will be mentioned.

Synchrotron facilities are uniquely equipped to handle space and time‐resolved SWAXS experiments, in contrast to benchtop SWAXS instruments. For example, spatially resolved SWAXS studies are primarily available using synchrotron sources, enabled by a sufficiently small, focused beam size [110, 111]. Similarly, time‐resolved SWAXS studies are possible with sufficiently high beam flux and low‐noise detectors [112, 113].

Whereas SWAXS uses relatively high‐energy X‐rays (>2500 eV), resonant soft X‐ray scattering (RSoXS) instead uses much smaller energies (<3 eV) as to match the specific absorption edges of different elements. The use of soft X‐rays enables the determination of two characterization features: highlighting isolated scattering contributions and determining orientational order [114, 115, 116, 117]. Firstly, isolated scattering contributions may be achieved by tuning the incident X‐rays to match the specific absorption edges of a specified element. As the electronic transition(s) associated with soft X‐rays are specific to each element, RSoXs serves as a useful characterization method to highlight the scattering contributions of the element. Secondly, toward determining orientational order, absorption of the incoming soft X‐ray radiation is maximized when the electric field vector of the incident X‐ray radiation is parallel to the transition dipole moment of the absorbing molecule. Linearly polarized soft X‐rays can then be used as a probe to determine the bond orientation of the molecule. The use of linearly polarized soft X‐rays is sometimes distinguished as near‐edge X‐ray absorption fine structure (NEXAFS) spectroscopy, and is typically carried out on soft matter samples, owing to the abundance of unidirectional bonds. In the context of LCs, RSoXS has recently been successful in deciphering many chiral nanostructures, including chiral smectic C phases, twist bend nematics, blue phases among others [118].

Recently, scanning SAXS has been combined with computer tomography to obtain a new characterization technique, small angle X‐ray scattering tensor tomography (SAS‐TT) [119, 120]. This method involves collecting thousands of detector images from a tomographic sample, and the reconstruction of the reciprocal space to give the 3D nanoscale information, as well as the orientation of each voxel. Previously, this technique has been limited by long experimental times (>24 h), however thanks to improvements to synchrotron instrument capabilities, Appel et al. was able to reconstruct a tomogram in 1.2 h [121].

Toward visualizing self‐assembled nanostructures, electron density maps were reconstructed using SWAXS data. While the execution is outside the scope of this article, we direct the readers to the following references detailing the reconstruction for centrosymmetric ordering [122, 123, 124].

## 3 SAXS of “Traditional” Liquid Crystals

**FIGURE 7:**
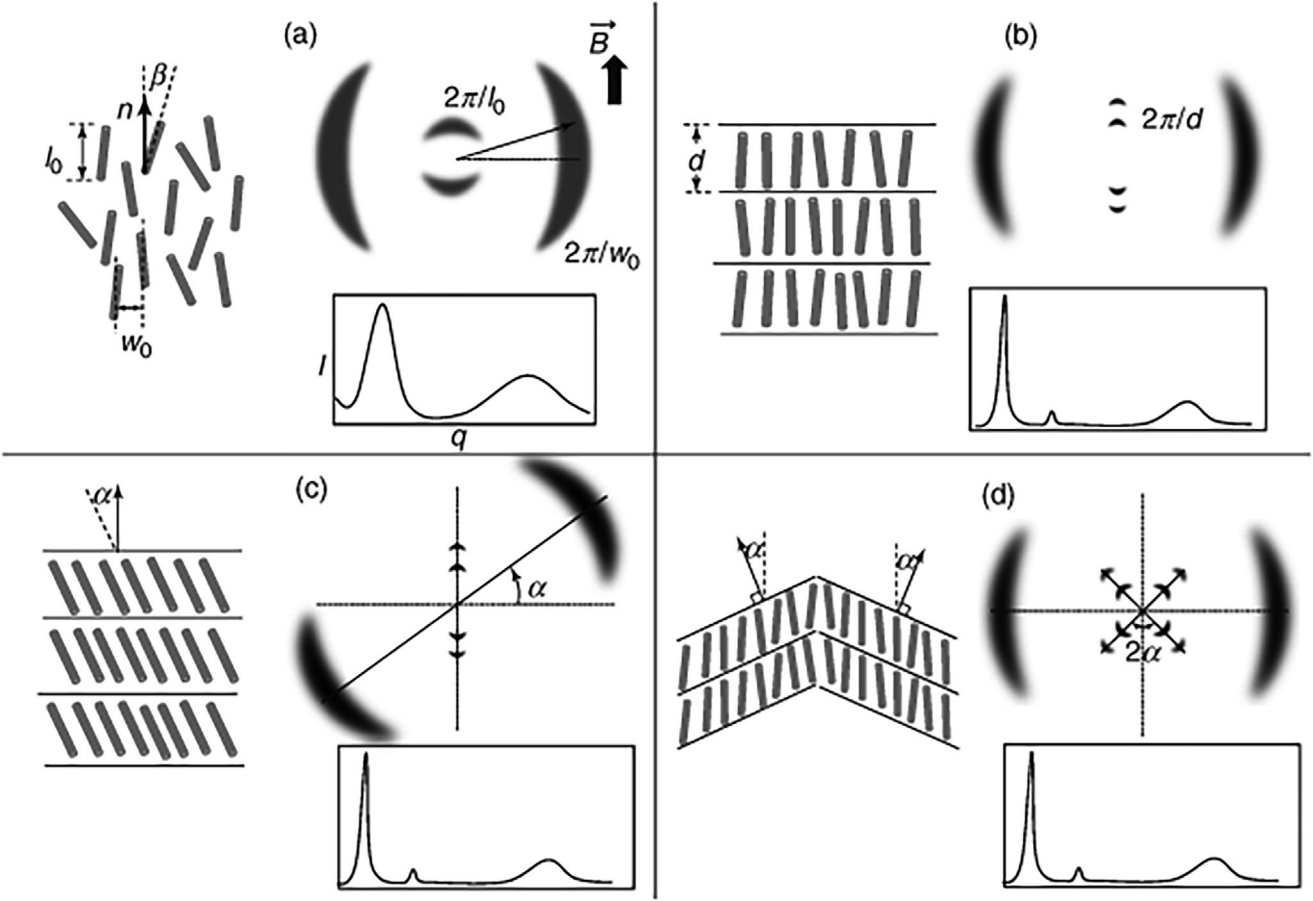
Schematic representation of scattering patterns resulting from common liquid crystals (LCs). (a) nematic, in this case, two expected diffused rings in the scattering patterns are expected at low and high q values, and these correspond to molecular length and diameter, *I*_0_ and *W*_0_, respectively, while *β* is the tilt angle. These diffused peaks are due to a lack of positional order. (b) SmA, a single domain in which *d* is the length of the Smectic layer (c) SmC, exhibits a tilt angle *α* in the single domain, d) Chevron pattern organization within SmC. Reproduced with permission [132]. Copyright 2014, Wiley.

LC possess many phases and herein we will only discuss the most encountered LC phases. A schematic diagram of these common aligned LCs and their resulting scattering patterns, is shown in Figure 7. Alignment methods include surface alignment/anchoring [125], flow alignment [126], application of a magnetic/electric field [127, 128], and drawing fibers [129, 130]. As can be seen, the scattering patterns are quite predictable, however, as this review will reveal, LCs rarely behave as ideal. For more detailed discussions regarding structure‐scattering phenomena (i.e. translational and orientational order, etc.) and more exotic LC phases (i.e. hexatic, incommensurate phases, and modulated LC phases etc.), we direct the readers to the following references [49, 93].

Owing to the nature of thermotropic and lyotropic LCs, the aforementioned linear fitting regions are rarely applicable to LC systems, as structure factors dominate. While most studies which utilize SAXS employ the form factor [8], most LC studies do not. In one exception, the form factor can be used to identify self‐assembled geometries, i.e., discs, rods, and flat bilayers [131]. For the purposes of identification, generally the form factor occurs at low *q* values and generally appears oscillatory, though this appearance is largely dependent on the molecules’ shape.

### 3.1 Nematic Liquid Crystals

Scattering of nematic LCs is generally quite simple to interpret, as X‐ray scattering arises due to its molecular dimensions. Since nematic LCs possess orientational order, with little positional order, this results in rather broad peaks. The major difficulty in analyzing nematic LCs is often the broadness of its signals and the length scales at which SAXS can probe.

**FIGURE 8:**
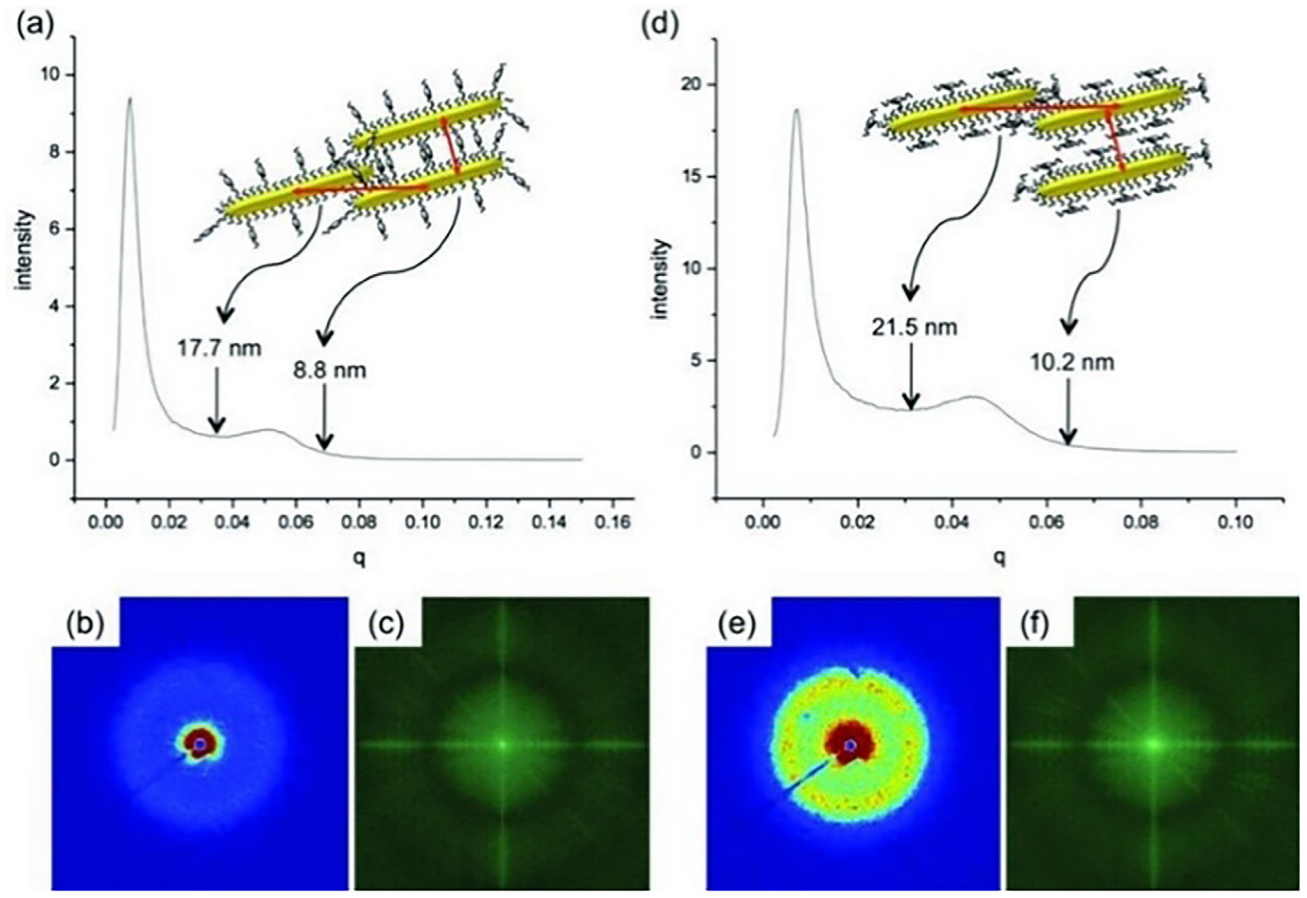
small‐angle X‐ray scattering (SAXS) scattering profiles for assembles of gold nanorods coated with end‐on nematic ligands (a–c), or side‐on nematic ligands (d–f). 1D plots reveal nanorod organization for end‐on and side‐on nematic ligands, in (a) and (d), respectively. 2D plots reveal an absence of long‐range organization due to isotropic scattering in (b) and (e). Fourier transformations of TEM images included in (c) and (f). Reproduced with permission [132]. Copyright 2013, Wiley.

One study carried out by Umadevi et al. explored films of self‐assembled gold nanorods (Figure 8) coated with either side‐on or end‐on nematic ligands [133]. The dimensions of their gold nanorods were 28.6 and 6.8 nm and their resulting films produced the concentric rings in their 2D profile (Figure 8b,e), which were azimuthally integrated into the 1D profiles in Figure 8a,d. The authors were able to nicely correlate the significant broad peaks observed with SAXS, to measurements obtained using high‐resolution TEM. They were able to determine the upper and lower limits of broad peak to be the longitudinal and side‐to‐side correlations between adjacent gold nanorods. Additionally, the authors reported smectic organization, but this was not observed in the SAXS, perhaps due to the infrequent occurrence of the smectic domains. Furthermore, the authors observe locally aligned domains in TEM with no overall long‐range alignment, which resulted in the concentric rings observed in the 2D pattern.

**FIGURE 9:**
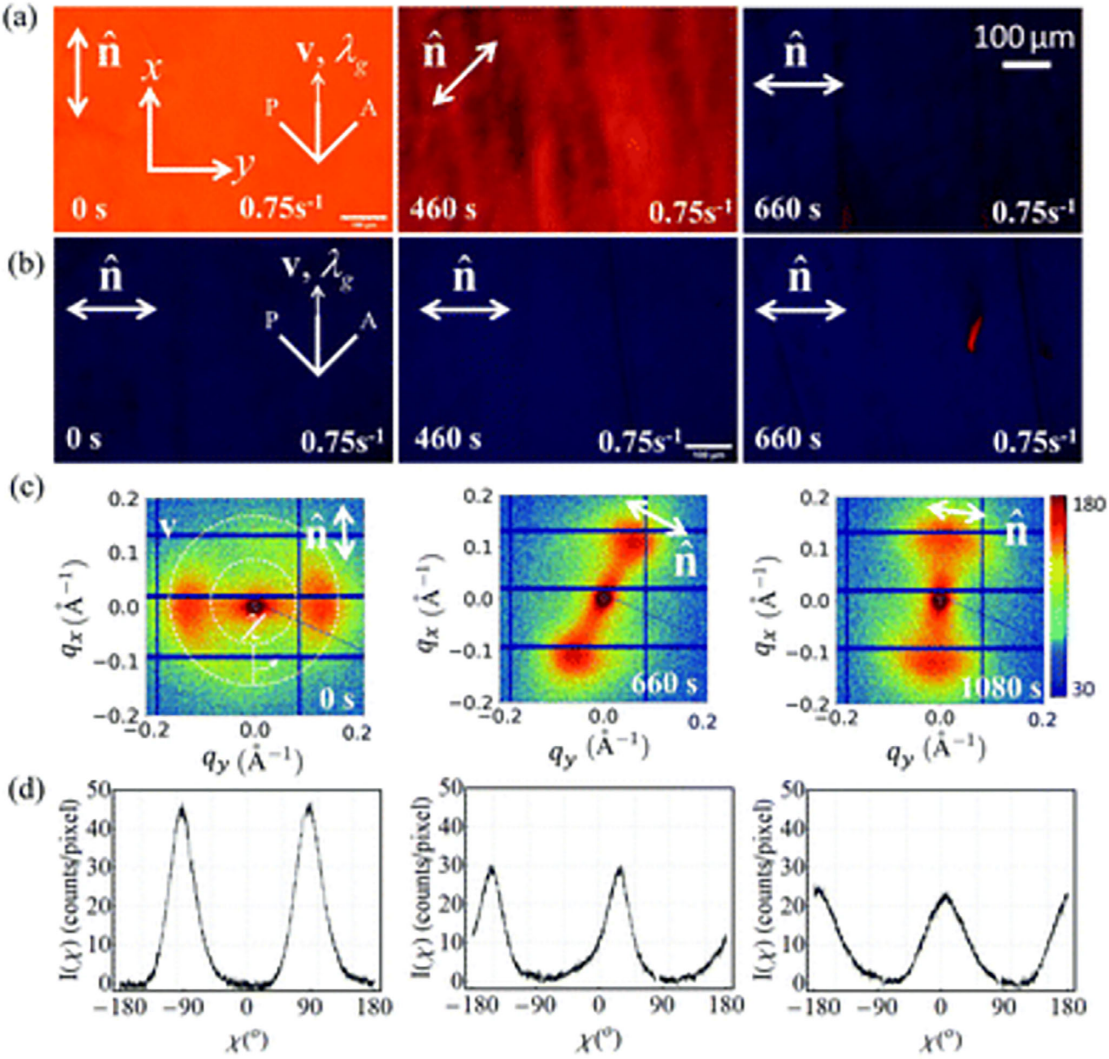
Polarized optical microscopy (POM) images of disodium cromoglycate (DSCG) when weak shear is applied (a) perpendicular to the director axis (denoted by n̂), and (b) parallel to the director axis, over 660 s. (a) Demonstrates reorientation of the nematic liquid crystal (LC) structure, while (b) maintains the director axis. 2D SAXS scattering profile of (a) is represented by (c) and its resulting azimuthal integration is depicted by (d). Reproduced with permission [134]. Copyright 2025, The Royal Society of Chemistry.

A rheological study conducted by Baza et al. demonstrates the time scale and re‐orientation of nematic LCs [134]. A nematic, disk‐like LC, disodium cromoglycate (DSCG), was subject to low to high shear, as to discern the role of shear in the tumbling mechanics of LC DSCG. Shear applied perpendicular to the director axis results in the reorientation of the DSCG, with equilibrium being fully achieved by 660s (Figure 9a). Reorientation of DSCG was tracked using SAXS, where the lateral reflections between the LC molecules were visualized in the 2D scattering profile. Azimuthal integration of the resulting pattern yields the distribution of orientations represented in a 2D profile, the calculated values confirm the reorientation of the DSCG by approximately 90°, as shown in Figure 9d.

**FIGURE 10:**
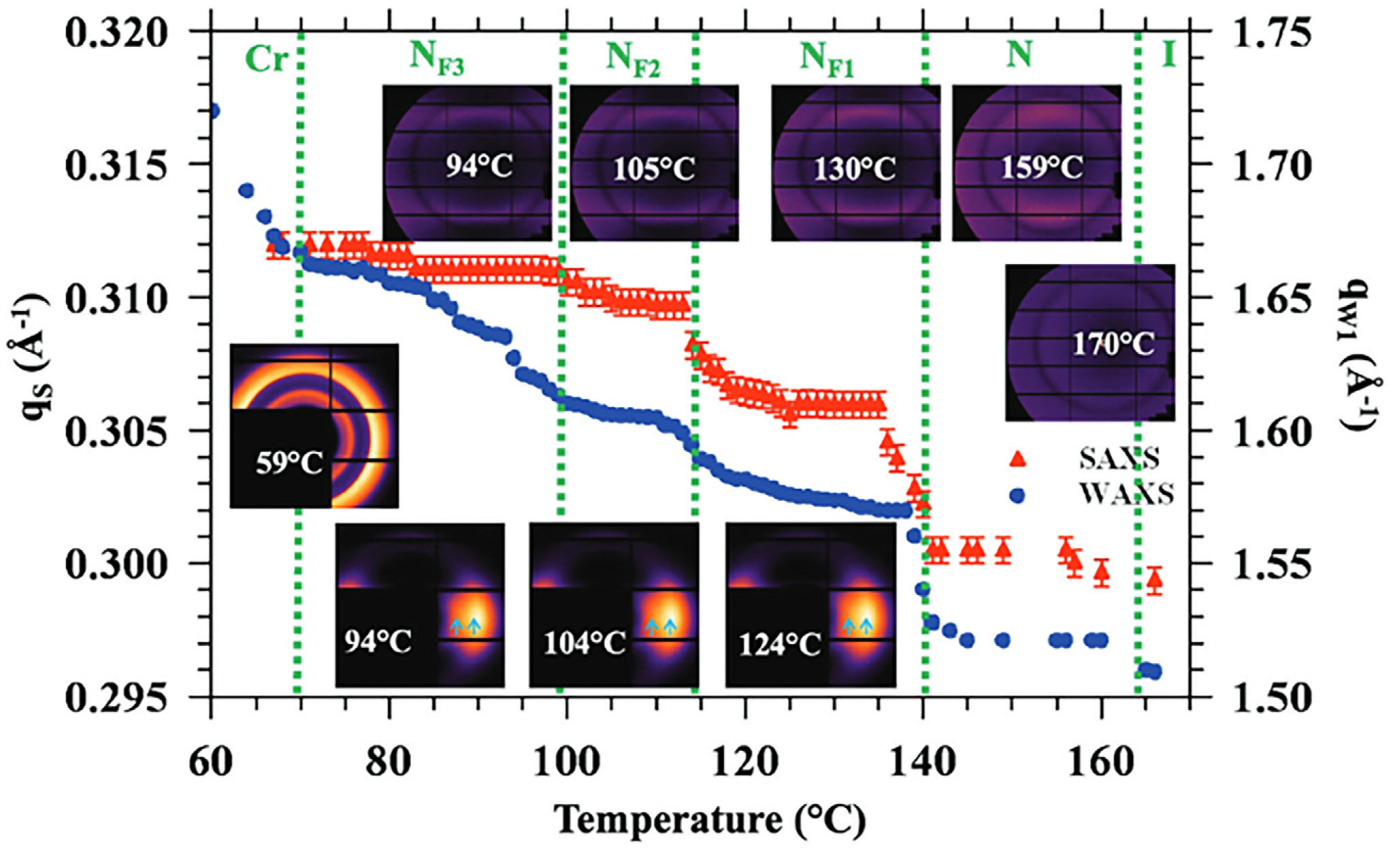
Phase diagram depicting the temperature transitions of the ferroelectric nematic liquid crystal (LC) phases of RT11001, as well as the shifting of the lateral correlations observed in small‐angle X‐ray scattering (SWAXS). SAXS pattern is depicted as insets in the upper right, and WAXS patterns are shown as insets in the bottom left. Reproduced under the terms of the CC‐BY‐NC‐SA 4.0 license [135].

In their study, Saha et al. were able to identify several new ferroelectric nematic phases, seen in their newly synthesized compound, 4‐nitrophenyl 4‐(2,4‐dimethoxylbenzoyl)oxy‐2‐fluorobenzoate, which they termed, RT11001 [135]. Their findings determined that the ferroelectric LC molecules, namely: N, N_F1_, N_F2_, and N_F3_, in order of decreasing temperature. The nematic cybotactic structure consists of molecularly packed aggregates dispersed within a nematic medium. Thanks to SWAXS, the authors were able to identify that two positional reflections were present, owing to the lateral correlation between neighboring RT11001 molecules within a nematic medium as well as reflections owing to a smectic cluster, indicating various nematic cybotactic LC structures. This is most evident in Figure 10, where one positional correlation is present in crystalline RT11001, but upon heating, two diffuse SWAXS peaks arise above ∼67°, indicating two LC domains (nematic and smectic). In conjunction with polarizability/relaxation studies, birefringence and DSC, the authors were also able to determine that each of these LC phases possessed combinations of polarities between the nematic medium and smectic clusters.

### 3.2 Cholesteric LCs

Popular depictions of cholesteric LCs portray the cholesteric structure as successive rotated planes of nematic layers. However, this depiction is primarily educational and the actual assemblies are closer to resembling a twisted spire, with a continuous twist [136, 137]. For this reason, cholesteric LCs are sometimes also known as twisted nematics. Since SAXS measures periodicity, successive twisted layers would produce significant Bragg diffractions, but this is not observed, giving further merit to a twisted nematic structure. Characterizing cholesteric LCs is generally the same as nematic LCs [132], due to the twisted nematic conformation, the cholesteric structure is orientationally ordered (about the helical axis), albeit with little periodicity, which manifests as broadened signals owing to each molecular dimension of the molecule.

**FIGURE 11:**
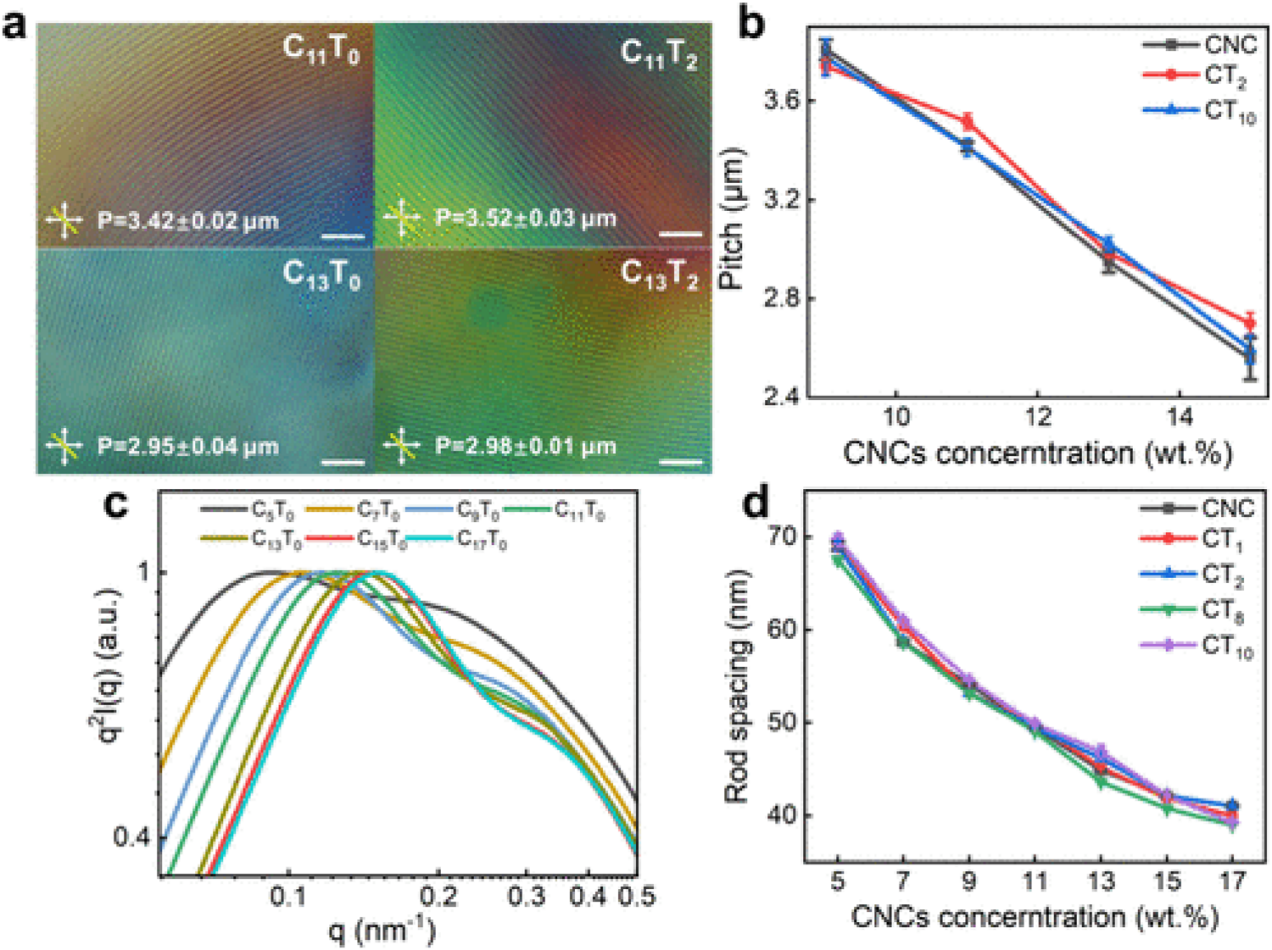
(a) Representative polarized optical microscopy (POM) images of the cholesteric cellulose nanocrystals/tannic acid composite mixtures. (b) Pitch measurements of cellulose nanocrystal (CNC)/TA mixtures were determined from POM. (c) Kratky plot of the cholesteric CNC/TA mixtures, which reveals the average interparticle distance. (d) Rod spacings as determined by Figure 7c. Reproduced with permission [138]. Copyright 2024, American Chemical Society.

Interestingly, Jie et al. studied the cholesteric behavior of composites of cellulose nanocrystals (CNCs) and tannic acid (TA) [138]. While the addition of tannic acid reduced viscosity and delayed gelation, the CNC structure was unaltered. However, the authors’ characterization and analyses were quite clever. They were able to measure the cholesteric twist angle by converting the 1D scattering profile to a Kratky plot, where the axes are transformed to *I*(*q*)*q* ^2^ vs. *q* (Figure 11). As a result, the interparticle distances were revealed as peaks, and the twist angle can be measured from Equation (17):

(17) $$\theta =360^{\circ }\;\times \frac{d}{p}$$

where *d* represents the interparticle distance measured from SAXS, and *p* represents the pitch, as measured from the cholesteric fingerprint pattern from POM. From their results, a clear trend can be seen where the interparticle distances decrease with increasing CNC concentration.

### 3.3 Smectic LCs

Since smectic LCs are the most “crystalline” of the LC phases, interpretation of the obtained SAXS data is typically straightforward. Observed reflections can be nicely indexed to the molecular dimensions of the LC molecule, as summarized in Figure 7b–d [49]. Additionally, owing to the periodicity of the smectic layers, several orders of diffraction may be visible. With regards to Figure 7b–d, two orders of diffraction are visible in the schematic, with the first order peak represented by the sharp, leftmost peak, and the second order peak represented by the sharp, but smaller intensity to the right. These reflections follow a 1:2 peak ratio, therefore the smectic phase can be characterized as lamellar structures (see Table 4). Furthermore, the additional broad peak at higher q represents the lateral correlations between the smectic molecules. The correlation length/smectic layer thickness of the LC can be determined through *d* = 2π/*q* of the primary peak. It is important to note that the correlation length does not necessarily represent the molecular length, as the layer spacing is included in the correlation length. While SAXS may easily elucidate the smectic structure, it is also possible to distinguish between smectic A from smectic C phases. This is most evident in the 2D scattering pattern, as the tilt angle arising from the smectic C phase appears as slanted peaks on the 2D patterns [49]. Furthermore, smectic layering arising from smectic C phases are smaller than smectic A phases, due to the tilt of the LC molecules within the layers.

**FIGURE 12:**
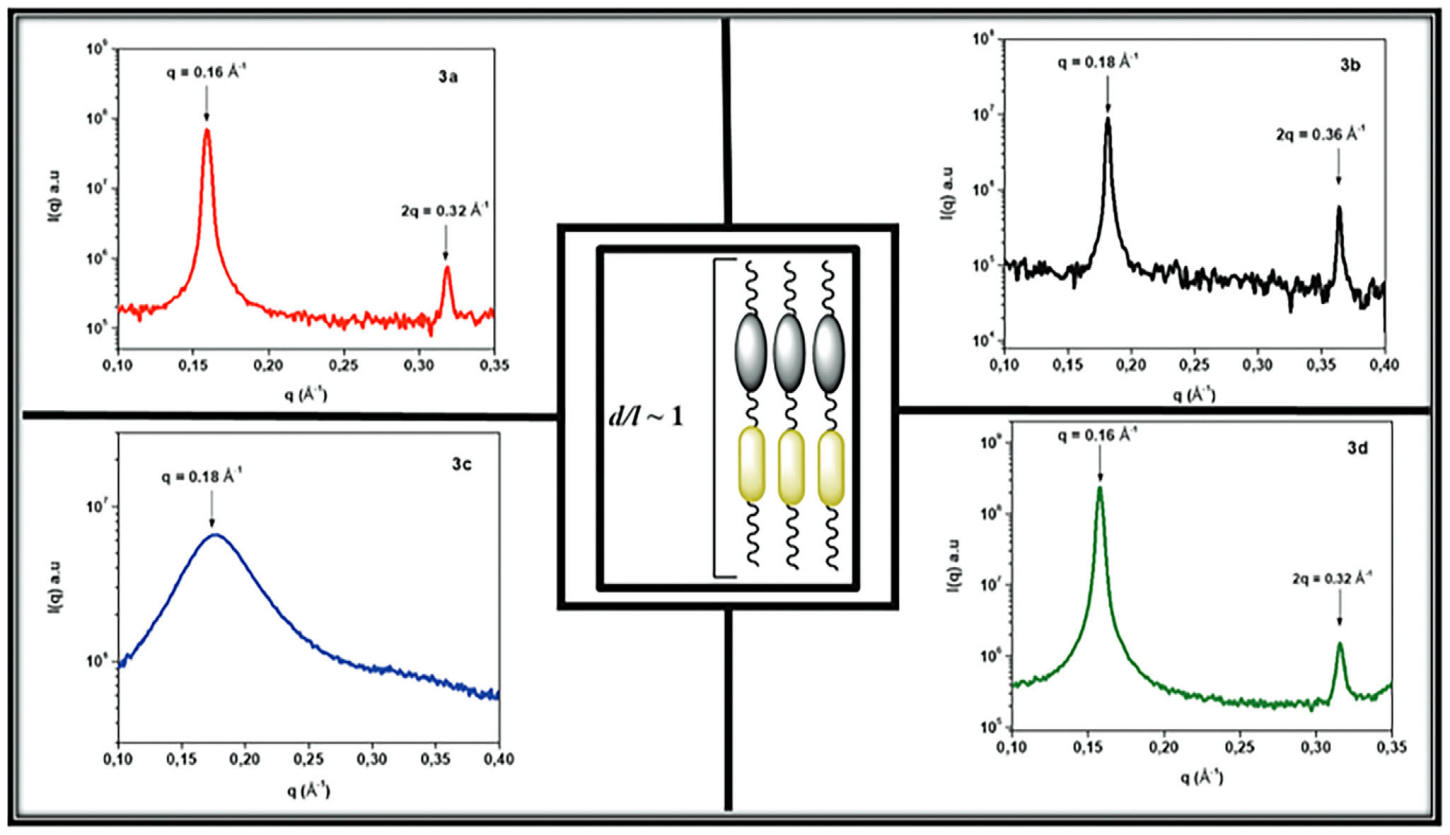
1D small‐angle X‐ray scattering (SAXS) profiles of various cholesterol containing liquid crystal (LC) dimers, which the authors labeled 3a‐3d. The unsymmetric dimers 3a,3b,3d exhibited smectic reflections, while 3c revealed potential cybotactic clusters. Reproduced under the terms of the CC‐BY 4.0 license [139].

Authors Frizon et al. report cholesterol‐containing LC dimers, which primarily formed smectic LCs (labeled 3a,3b,3d) with one synthesized dimer forming a cholesteric LC instead (3c) [139]. Characterization of the smectic LCs was relatively straightforward, as the dimers clearly exhibited two orders of diffraction, with a clear 1:2 peak ratio giving a lamellar arrangement (Figure 12 and Table 4). Obtaining the real distance for each of the smectic dimers results in the smectic layer thickness. The authors represented the smectic layer thickness to the molecular length of the dimers as a ratio (*d/l*) as to determine the composition of each layer. Since *d/l* was determined to be ∼1, the authors concluded that each layer was composed of one dimer laid perpendicular to the layer direction. In the case of the dimer, 3c, a significant broad peak exhibiting first‐order diffraction was observed. The authors hypothesize that this is due to cybotactic smectic clusters within a nematic medium, which is common for bent core mesogens [140]. While it is possible, the alternate explanation is that 3c simply forms a cholesteric LC instead. Polarized optical microscopy revealed perhaps the onset of a focal conic texture, suggesting a cholesteric LC. Furthermore, it is likely that the broad peak observed in SAXS corresponds to the correlation length of a cholesteric LC, with poorly defined layering. The *d/l* was also determined to be 0.8, indicating that there is lengthwise intercalation between dimers. It was not mentioned whether the SAXS samples were aligned, but alignment could aid in the confirmation of cybotactic clusters.

**FIGURE 13:**
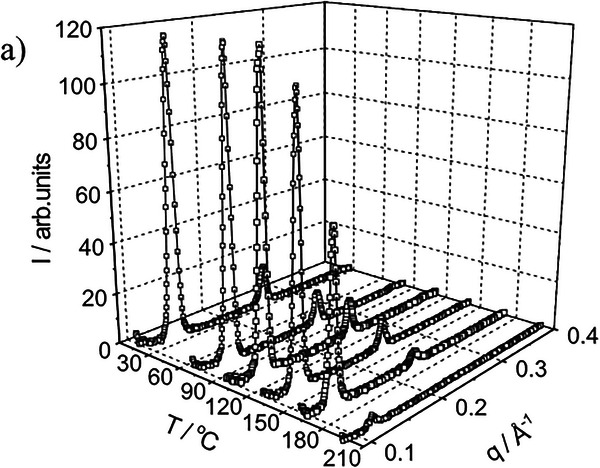
Temperature dependence of a side‐chain liquid crystalline polyacrylate with a four‐carbon spacer and eight‐carbon tail, P_48_. The sample undergoes g‐SmC‐SmA‐I transitions at 58°C, 130°C, and 155°C, respectively, which is evident in the 1D spectra. Reproduced under the terms of the CC‐BY NC 4.0 license [141].

Pereira et al. conducted an elegant analysis of various polyacrylates with LC side chains [141]. The authors studied a total of four side‐chain liquid crystalline polyacrylates, with varying spacer lengths and terminal tails, which resulted in thermotropic LCs which possessed either g‐SmA‐I or g‐SmC‐SmA‐I transitions, where glassy and isotropic states are represented by *g* and *I*, respectively. The scattering profile of one such polymer is presented in Figure 13, possessing a four‐carbon spacer and an eight‐carbon tail, which the authors labeled P_48_. P_48_ undergoes a g‐SmC transition at 58°C, a SmC‐SmA transition at 130°C and a SmA‐I transition at 155°C. These transitions are immediately evident in the SAXS profile; with a slight shift toward higher *q* values between 120°C and 150°C, and diminished intensities above 150°C, indicating reduced periodicity, a.k.a. crystallinity. Furthermore, two orders of diffraction are observed, with q values obeying a ratio of 1:2, which can be attributed to the smectic layering. The authors note that due to the non‐preferential alignment of P_48_, the tilt angle could not be elucidated from the 2D scattering pattern. However, the tilt angle can still be calculated trigonometrically using the layer spacings and Equation (18):

(18) $$\theta =\cos^{-1}\frac{d_{smC}}{d_{smA}}$$

which was determined to be 22.5° for P_48_. The authors were also able to determine that each layer was comprised of slightly intercalated LC side chains, as the layer thicknesses were 1.65 times the length of a single LC side chain group, suggesting a slight overlap between the groups within the smectic layer.

**FIGURE 14:**
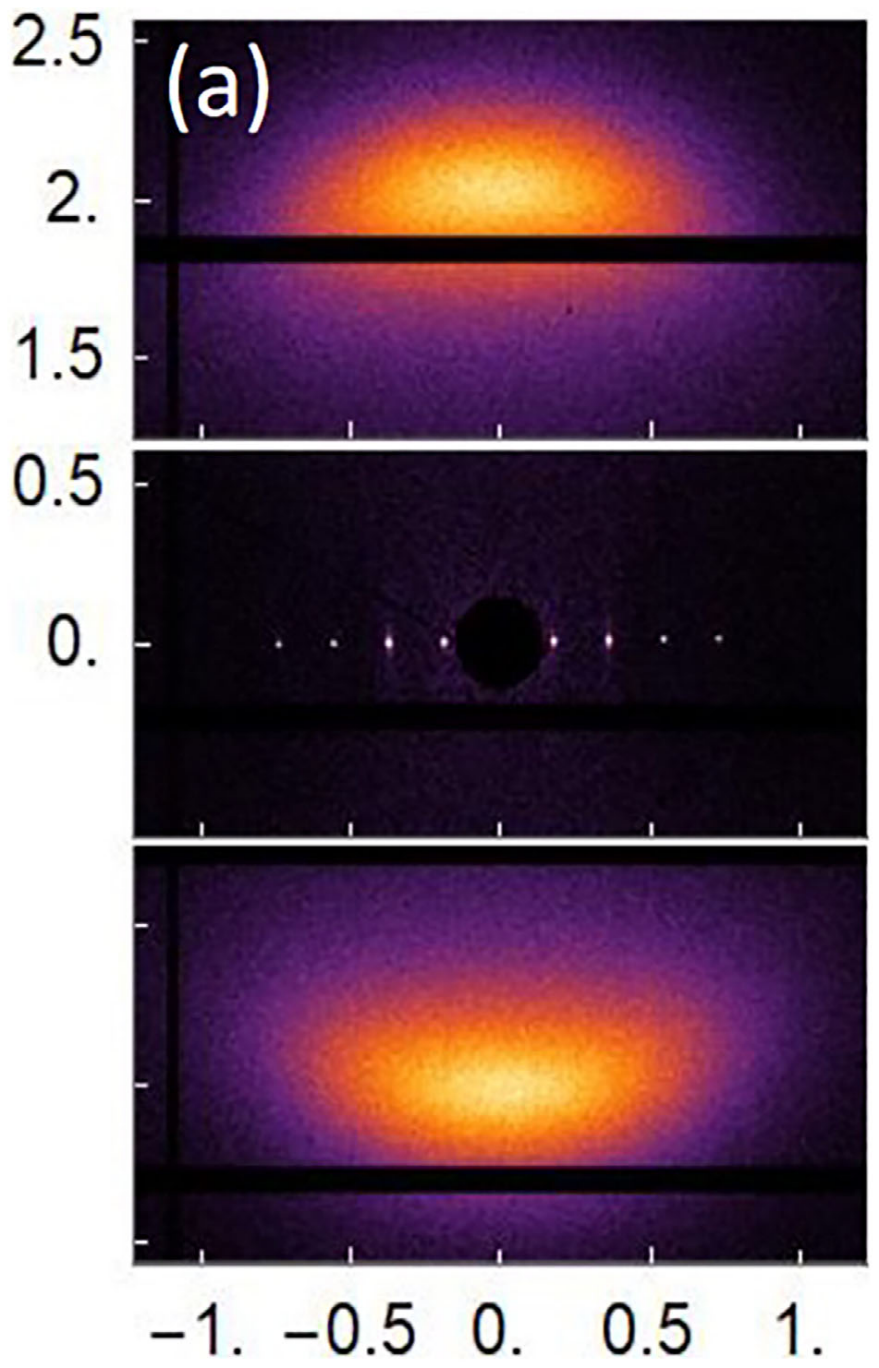
2D small‐angle X‐ray scattering (SAXS) pattern obtained from DNA duplex, 48‐20T‐48. Two 48‐base pair duplexes were connected by a single stranded segment comprised of 20 thymine bases. Reproduced under the terms of the CC‐BY 4.0 license [142].

Similarly, Gyawali et al. were able to produce highly aligned, single‐domain samples of DNA duplexes [142]. The authors revealed that samples of DNA segments of variable lengths connected with a single‐stranded thymine bridge were able to produce both cholesteric and SmA LC phases. The resulting spectra of one representative SmA sample is shown in Figure 14; here, the sample was comprised of two 48‐base pair duplexes connected with a thymine bridge comprised of 20 units and was deemed 48‐20T‐48. Significantly, two sets of orthogonal reflections, representing the directionality of the smectic features. A large, diffuse pair of vertical reflections at 1.9 nm^−1^, or 3.3 nm^−1^, arises due to the lateral spacings between the DNA duplexes. In contrast, sharp peaks across the horizontal direction represent four orders of diffraction owing to the thickness of the smectic layers. The first order reflection at 0.18 nm^−1^ corresponds to a layer thickness of 34.5 nm, which in turn can be indexed to be slightly larger than the length of two 48‐base pair duplexes (32 nm). This suggests a smectic bilayer, where each layer is comprised of pairs of duplexes arranged end‐to‐end.

Chen et al. were able to successfully synthesize a thermotropic chiral luminescent LC (NO_2_‐CS‐C_6_‐Chol) and were able to observe its transition from crystalline, to SmC, to cholesteric, using SAXS [143]. In the crystalline phase, several distinct diffraction peaks are visible below 130°C, owing to the crystalline structure. Upon heating, the resulting 1D profile reveals two diffraction peaks owing to the SmC structure, with the layer spacing calculated to be 6.28 nm (from 2π/*q*). The diffraction peaks disappear beginning at 155°C, while the fingerprint texture is observed using POM, confirming cholesteric behavior. Unfortunately, the authors didn't measure the tilt angle of the SmC structure.

**FIGURE 15:**
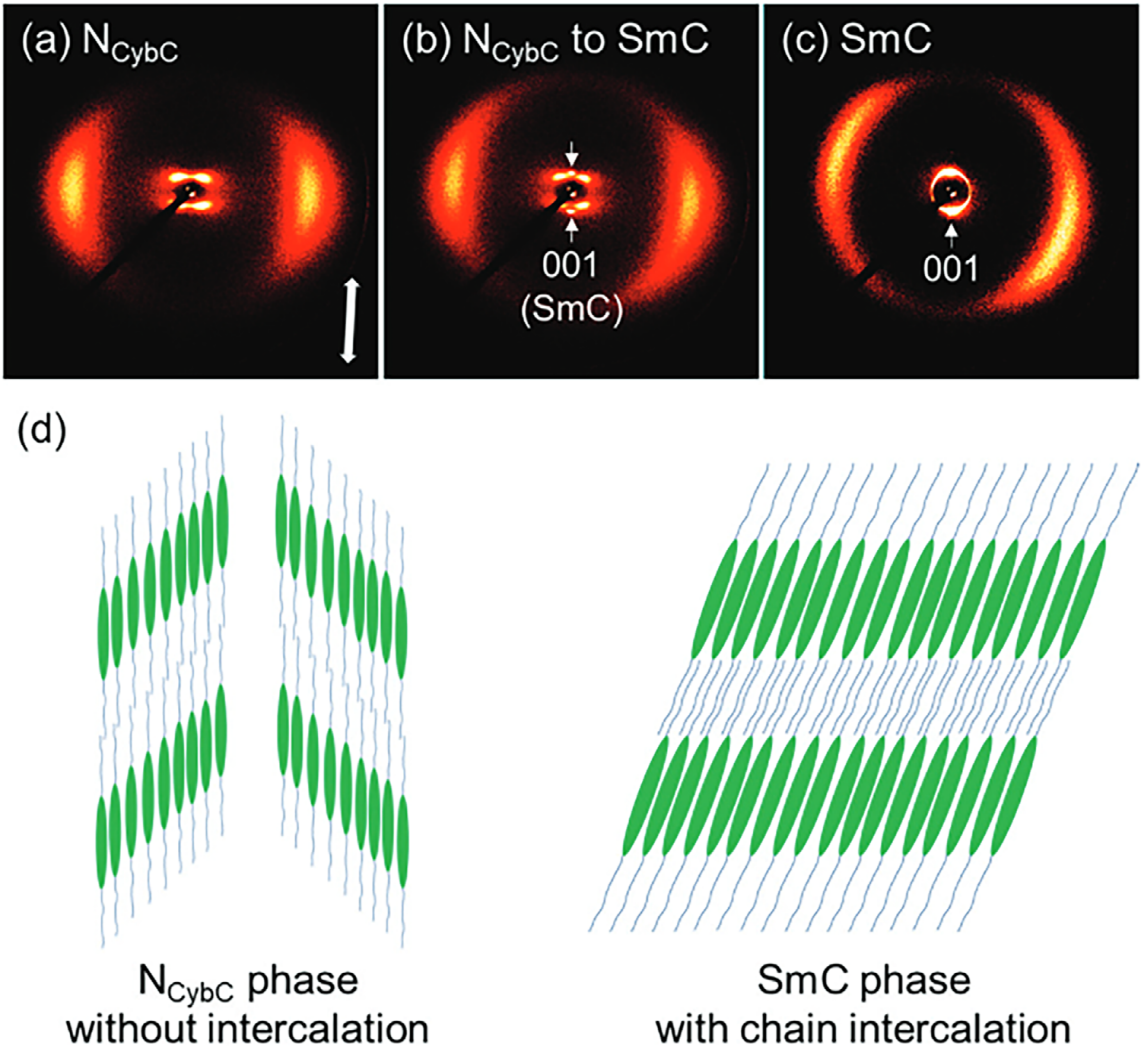
Small‐angle X‐ray scattering (SWAXS) pattern of SO8 exhibiting a transition from N_CybC_ to SmC at (a) 140°C, (b) 130°C, and (c) 120°C. (d) Schematic representation of the N_CybC_ and SmC LC phases. These experiments were carried out in 15 mm capillaries sandwiched between two pairs of magnets; hence, WAXS data were collected in transmittance mode. The magnetic field direction (and the long molecular axis orientation) is denoted by the white arrow in (a). Reproduced under the terms of the CC‐BY NC 4.0 license [144].

A novel mesogen with alkylthio and alkoxy end groups (SO8) was synthesized and characterized by Arakawa et al. [144]. Upon application of a magnetic field, a distinct transition between nematic cybotactic C (N_CybC_) and SmC is clearly observed in SAXS. The observed N_CybC_ depicts a dumbbell‐like pair of peaks in the small‐angle region (Figure 15a), which represents the lateral positional correlations between tilted nematics, while each “half” of the dumbbell represents the two present tilt angles. The measured tilt angle is 43° indicating that the nematic phase is comprised of “short‐range tilted SmC‐like pseudo‐layer clusters.” Upon cooling, SO8 adopts a fully SmC arrangement (Figure 15c), with only one tilt angle (∼20°) observed. Furthermore, the authors reason that there is also a contraction of the alkyl chains upon cooling, resulting in intercalation in the SmC phase, as evidenced by the smaller observed d‐spacings.

## 4 Amphiphilic Liquid Crystals

SWAXS is also suitable for elucidating the structures of amphiphilic compounds. Amphiphilic compounds are comprised of two distinct phases, typically hydrophobic and hydrophilic, which self‐assemble into a diverse library of nanostructures [145]. This phenomenon is commonly seen in amphiphilic lipids/surfactants, as well as amphiphilic copolymers. In contrast to the LCs mentioned in the previous section, assemblies generated from amphiphilic molecules often form cubic phases [146]. As cubic phases are optically isotropic, and therefore not birefringent, POM can easily distinguish them from non‐cubic LC phases, but cannot distinguish between various cubic phases, thereby rendering SWAXS and XRD essential in identifying and characterization of these amphiphilic assemblies.

**FIGURE 16:**
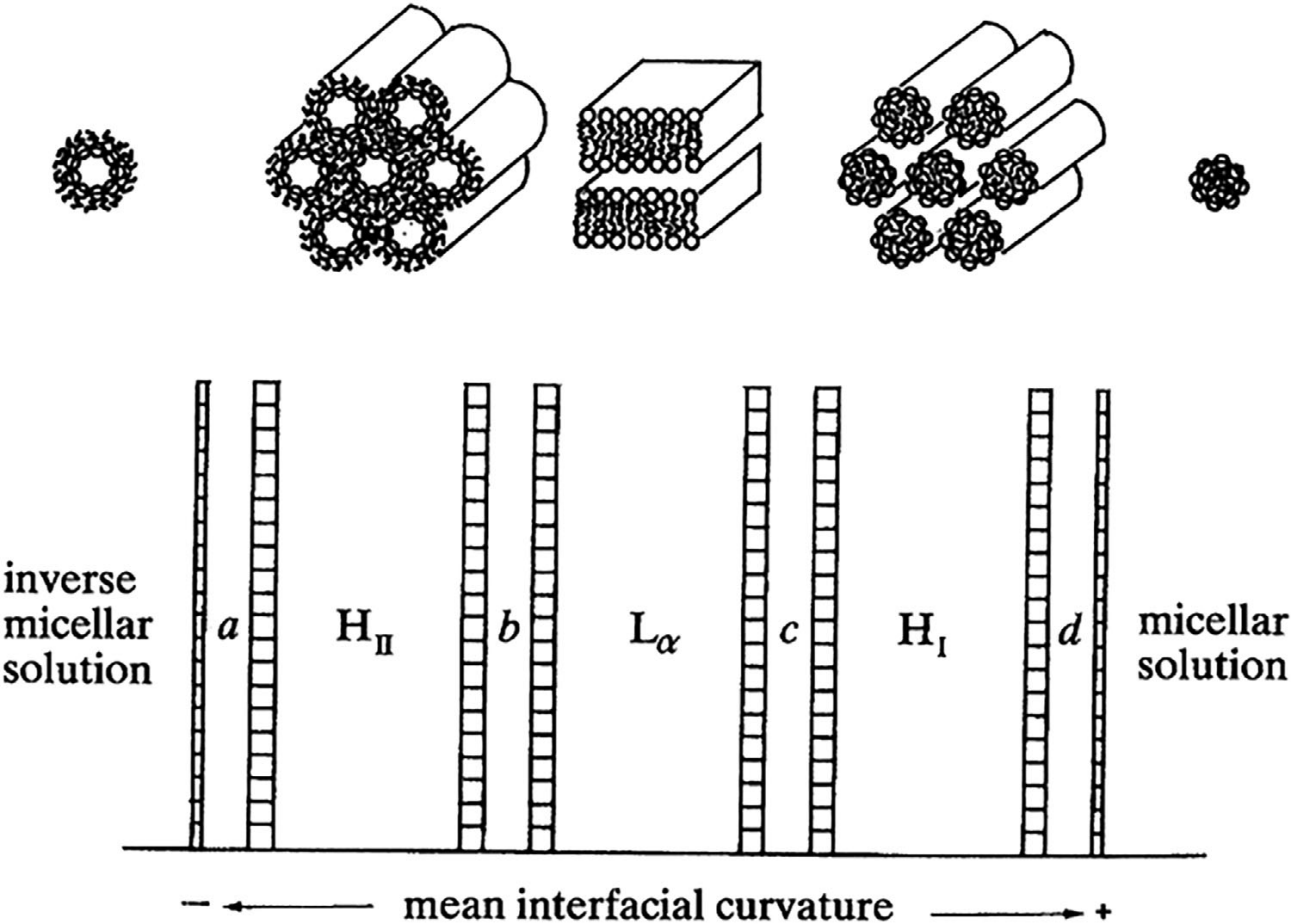
Spectrum of self‐assembled amphiphilic assemblies in terms of mean interfacial curvature. Depicted are the discontinuous phases, from left to right: inverse micellar, inverse hexagonal (H_II_), lamellar (L_α_), hexagonal (H_I_), and micellar. The transitional phases are non‐specific, and are denoted as (b) and (c), which are the bicontinuous cubic phase ranges VII (b) and VI (c) and a, d, which are the micellar cubic ranges I_2_ (a) and I_1_ (d). Used with permission of The Royal Society UK, Physical sciences and engineering, ROYAL SOCIETY (GREAT BRITAIN), 1990; permission conveyed through Copyright Clearance Center, Inc., 2025 [150].

**FIGURE 17:**
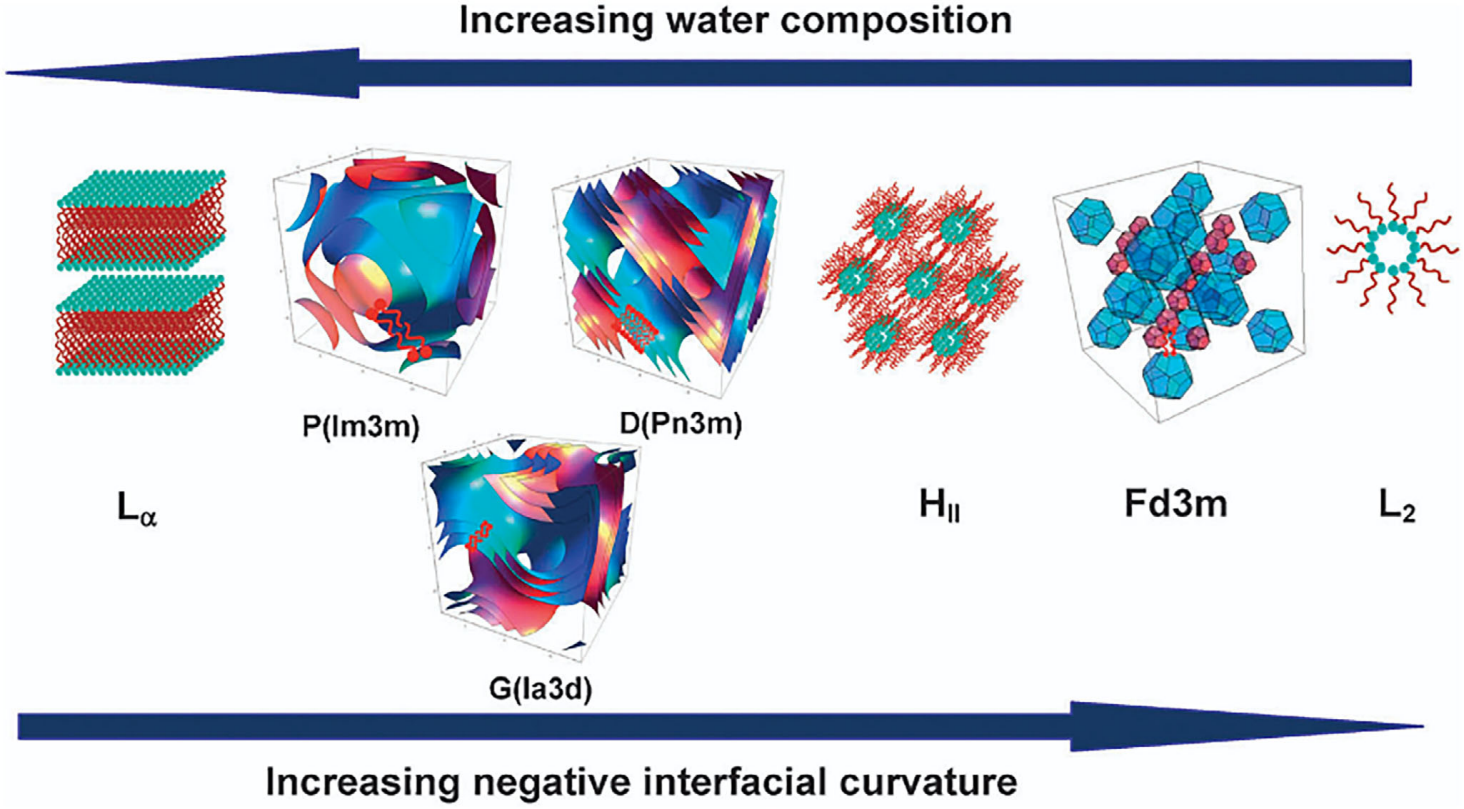
The “natural” sequence of amphiphilic self‐assembly in order of increasing negative interfacial curvature. From left to right the phases depicted are: lamellar (L_α_), primitive (*Im* $\bar{3}$ *m*), gyroid (*Ia* $\bar{3}$ *d*), diamond (*Pn* $\bar{3}$ *m*), hexagonal (H_II_), discontinuous micellar cubic (*Fd* $\bar{3}$ *m*), and inverse micellar (L_2_). Reproduced with permission [150]. Copyright 2025, The Royal Society of Chemistry.

Owing to the immiscibility of the hydrophobic and hydrophilic phases, complex molecular assemblies arise due to the hydrophobic effect, the tendency for hydrophobic groups to aggregate when dispersed in water [147, 148]. These self‐assembled structures are typically distinguished as either type I or type II, depending on whether the curvature of the interface between the two phases curve away from, or toward the aqueous phase, respectively. Furthermore, type II phases are also sometimes referred to as inverse phases. Figure 16 depicts the spectrum of amphiphilic assemblies in terms of mean interfacial curvature [149]. From left to right, the sequence can be thought of as an increasing fraction of water within the amphiphilic system, with the left side of the spectrum representing a negative curvature (bending toward water) and the right side representing a positive curvature (bending away from water). The major phases include the lamellar phase, L_α_, which lies in the center and possesses a flat, zero‐curvature interface, alongside the hexagonal and micellar phases (with their inverse counterparts). The in‐between phases (denoted b, c) are transitional phases unique to the system being examined, but are most commonly bicontinuous cubic phases, with the most common being primitive (*Im* $\bar{3}$ *m*), diamond (*Pn* $\bar{3}$ *m*), and gyroid (*Ia* $\bar{3}$ *d*). While phases (denoted a and d) represent micellar cubic phases, with the most common being body‐centered (*Im* $\bar{3}$ *m*), face‐centered ($Fn\bar{3}m$ and $Fm\bar{3}m$), and the so‐called A15 phase (*Pm* $\bar{3}$ *n*) [149]. Figure 17 represents the “natural” sequence of amphiphilic self‐assembly, in order of increasing negative interfacial curvature [150].

SWAXS and XRD are highly effective for characterizing amphiphilic assemblies because their high symmetry produces intense, well‐defined scattering signals. Identification of the specific LC structure can be accomplished by analyzing the characteristic peak ratios, as discussed in Section 2.7, and a list of the most encountered LC structures (and their characteristic peak ratios) is available in Table 4. As far as X‐ray based characterization, it is important to note that type I phases are indistinguishable from their type II counter parts. For example, the H_I_ and H_II_‐phases both exhibit the same characteristic peak ratios of 1:√3:√4:√7:√9.

**FIGURE 18:**
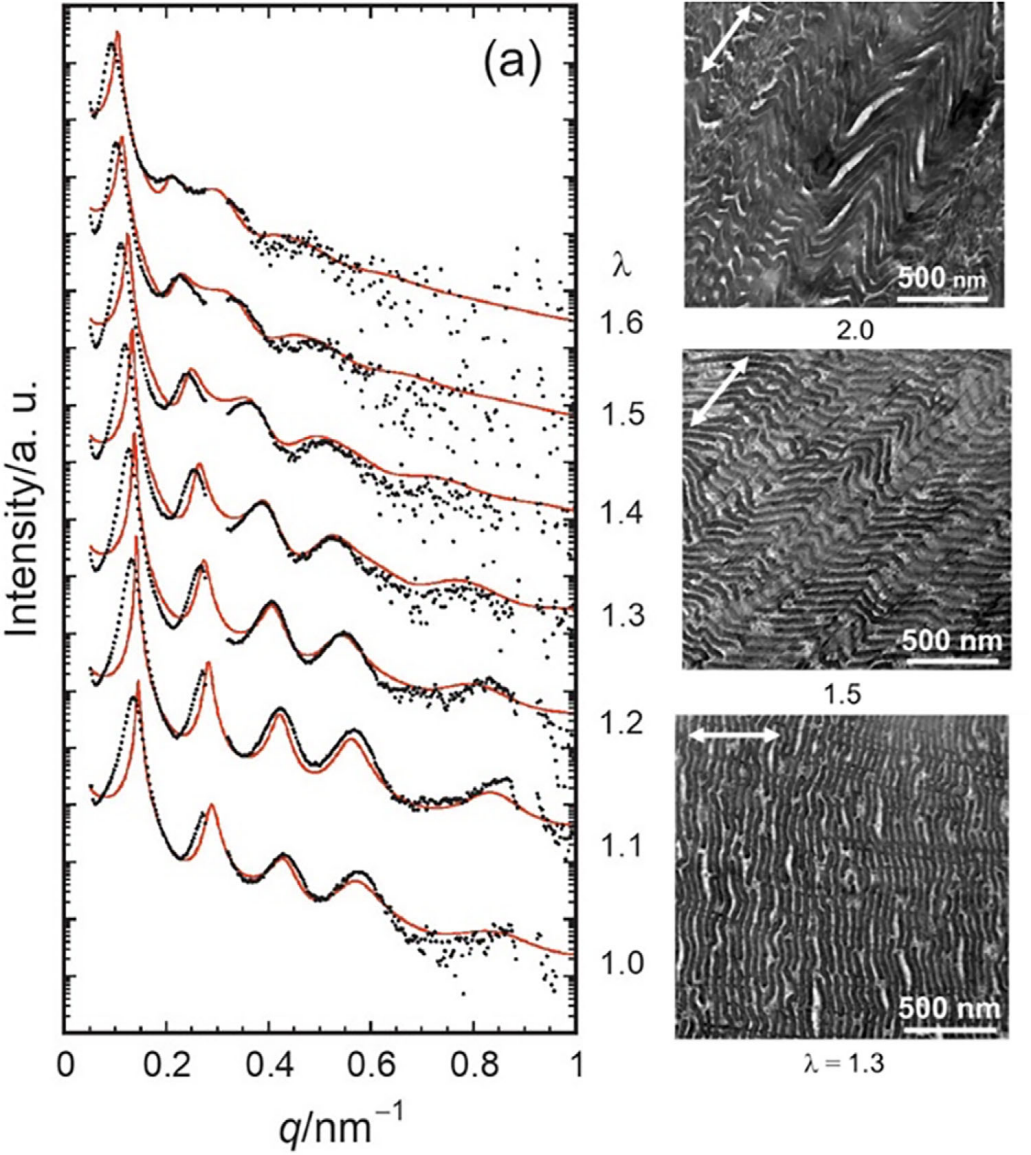
(Left) Small‐angle X‐ray scattering (SAXS) intensity profiles of an liquid crystal (LC) block copolymer as the elongation ratio, *λ*, is increased. Note that the solid red line represents the calculated intensity based on the ideal two‐phase lamellar model. (Right) Representative TEM images of the LC block copolymer at various elongation ratios. Reproduced with permission [151]. Copyright 2020, Wiley.

An example of a lamellar self‐organization is studied by Kuribayashi et al. [151]. Here, fibers of a LC block copolymer comprised of a central smectic LC polyester block which was enclosed by two poly (ethyl methacrylate) domains, which resulted in a lamellar morphology upon thermal annealing. To study the deformation of the lamellar microstructure, the authors loaded the fibers with weights and recorded SAXS measurements at various elongation ratios, λ. The 1:2:3:4 peak ratio characteristic of a lamellar phase was clearly visible at low λ, however as λ increased, the signal clearly decayed, indicating rippling of the lamellae, as seen in the TEM images (Figure 18).

**FIGURE 19:**
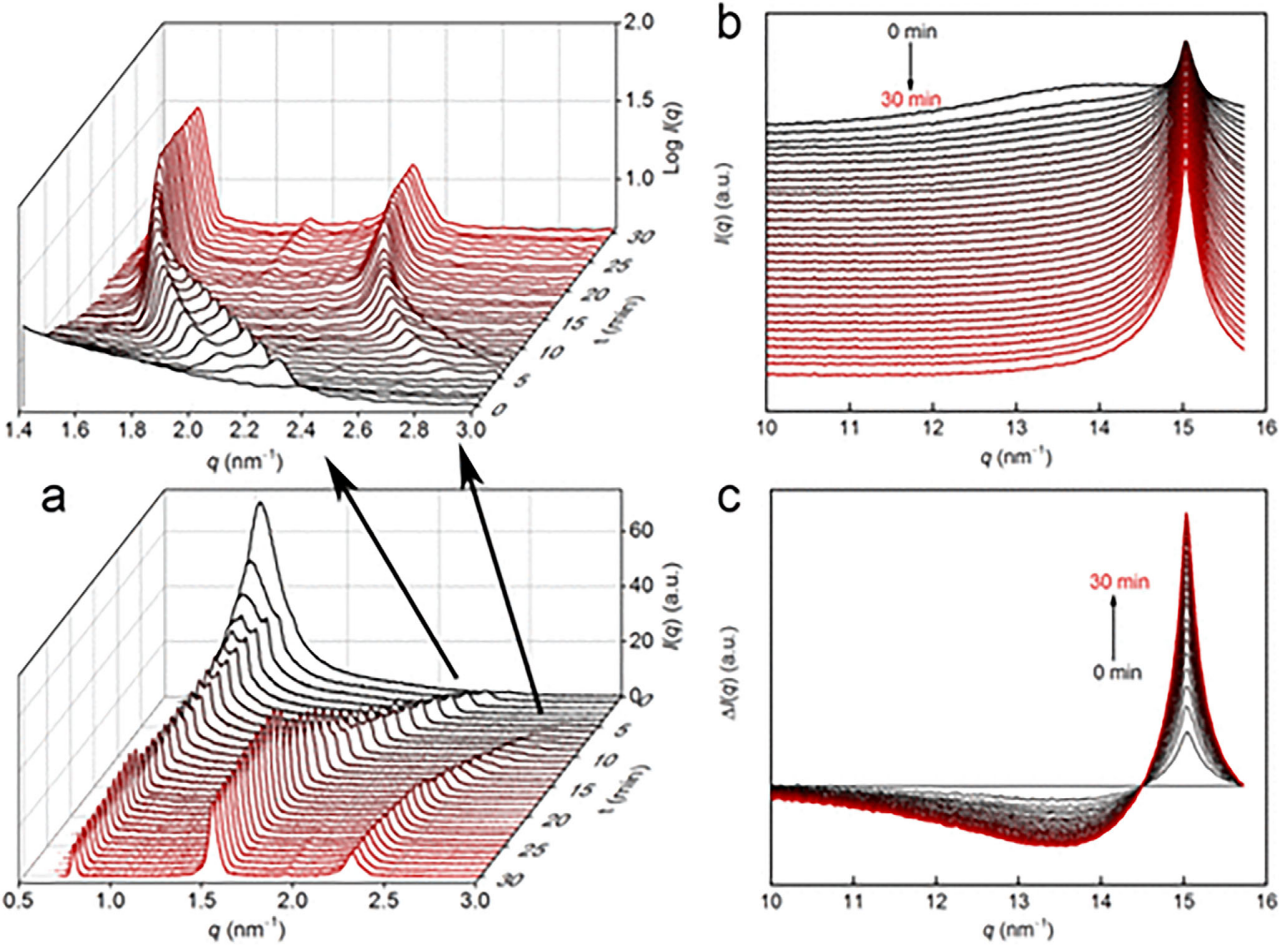
Small‐angle X‐ray scattering (SAXS) data obtained from C_16_mimCl. (a) Time‐resolved SAXS profile of the phase transition from micellar to lamellar structure in C_16_mimCl. (Top) Inset reveals the presence of two sets of lamellar structures converging toward a singular structure. (Bottom) Full scale SAXS profile. (b) WAXS region revealing disorder‐to‐order transition in the alkyl chain packing. (c) Resulting difference spectra of Figure 19b, by subtracting each subsequent spectra by the spectra obtained at 0 min. Reproduced with permission [152]. Copyright 2025, American Chemical Society.

Cao et al. studied the micellar to lamellar phase transitions of 1‐hexadecyl‐3‐methylimidazolium chloride (C_16_mimCl), an amphiphilic molecule [152]. The phase transition was tracked by an initial quick temperature drop from 30°C to 6°C, and SAXS measurements were obtained at periodic time intervals. Here, the authors were able to clearly see the 1:2:3 ratio, characteristic of lamellar structures, emerge from a single, broad peak corresponding to “intermicelle interference” (Figure 19). In general, micelles in solution exhibit a singular peak (representing the planes formed from the antipodes of the micelle), however, concentrated micelle solutions instead exhibit the BCC or FCC peak ratios [153, 154]. Furthermore, the high‐resolution spectra the authors obtained at the synchrotron revealed the presence of two sets of lamellar structures as an intermediate (Figure 19a (top)) before converging to a singular lamellar structure. Additionally, alkyl chain packing was observed to undergo a disorder‐to‐order transition in the WAXS region; as the sample quickly cooled, the peak observed at 15.03 nm^−1^ was significantly sharpened, while a broad peak centered about 14.01 nm^−1^ disappeared over time. Generally, transitions of this type are easily observed using SAXS, owing to the sharp interface arising due to microphase separation between the hydrophobic/hydrophilic regions [155].

It is essential to note that analyzing the crystal lattice is a crucial step that can facilitate the identification of multiple coexisting phases. As an alternative to characteristic peak ratios, some authors index the observed peaks through traditional crystallographic methods. An illustrative example was reported by Cho and co‐workers, in which they synthesized an amphiphilic compound (AB_2_) comprising 2,6‐dimethyl octyl chains and triethylene oxide (TEO) functionality [156]. Due to the short chains employed for crystallization, electrolytes Li+/ethylene oxide = 0.2 were used, leading to the formation of the hexagonal columnar phase at room temperature, which were attributed to the observed (100), (110), (200), and (210) planes. However, upon heating, a new distinct set of peaks, (220), (321), (420), and (332), appeared beginning at 50°C, which were indexed to the bicontinuous cubic group, *Ia*–$\bar{3}$ *d*. This suggests a simultaneous phase coexistence of the hexagonal columnar and the *Ia* $\bar{3}$ *d* bicontinuous cubic structures at a temperature range between 50°C and 70°C. Upon further heating, the two phases subsequently became completely disordered at 80°C.

**FIGURE 20:**
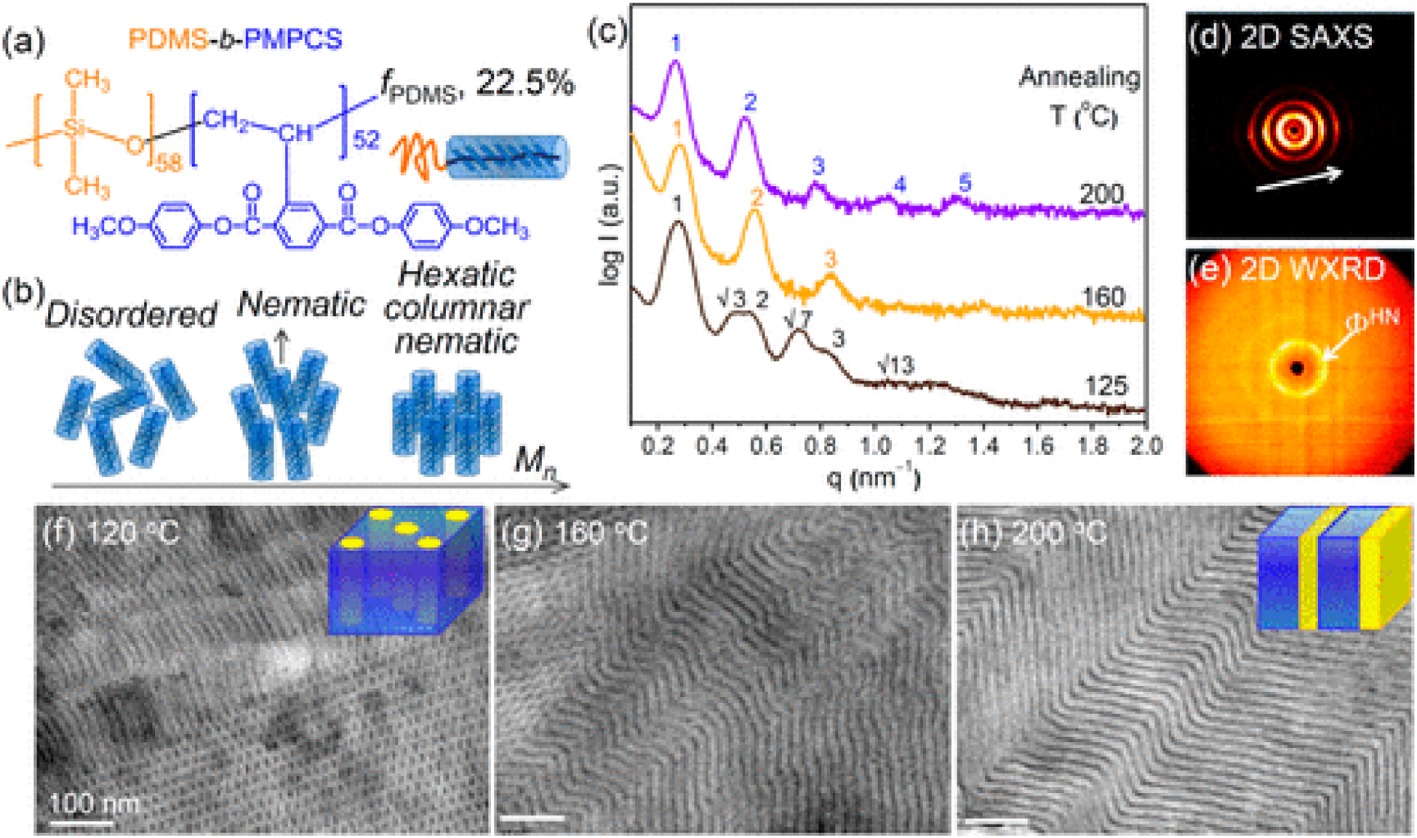
(a) Chemical structure of a liquid crystalline block copolymer, abbreviated as PDMS‐b‐PMPCS. (b) Schematic representation of the tendency of high molecular weight samples of PDMS‐b‐PMPCS to form hexatic columnar liquid crystal (LC) phases. (c) Small‐angle X‐ray scattering (SAXS) intensity profile representing the phase transition from hexagonal columnar at 125°C to lamellar at 200°C. (d,e) 2D SAXS and WAXS patterns of the lamellar PDMS‐b‐PMPCS at 200°C. (f–g) TEM images of samples representative of the results obtained in Figure 20c. Reproduced under the terms of the CC‐BY 4.0 license [157].

A block copolymer, PDMS‐b‐PMPCS, was revealed to undergo a clear hexagonal to lamellar transition in a study carried out by Shi et al. [157]. When initially annealed at 125°C, the samples of PDMS‐b‐PMPCS revealed a hexagonal morphology, which was immediately evident in Figure 20c. However, thermally annealing the sample at higher 160°C and 200°C instead produced a zigzag lamellar morphology. Using SAXS, the authors were able to easily discern both structures. The SAXS profile at 120°C revealed several reflections comprising the characteristic peak ratio 1:√3:√4:√7:√13, which corresponds to a hexagonal columnar structure. Additionally, the periodicity of the structure was measured to be 24 nm, as the primary peak occurs at 0.260 nm^−1^. At 160°C, a 1:2:3 peak ratio was observed, corresponding to a lamellar morphology to three orders of diffraction. Further thermal annealing resulted in improved organization through microphase separation, where five orders of diffraction were observed instead.

**FIGURE 21:**
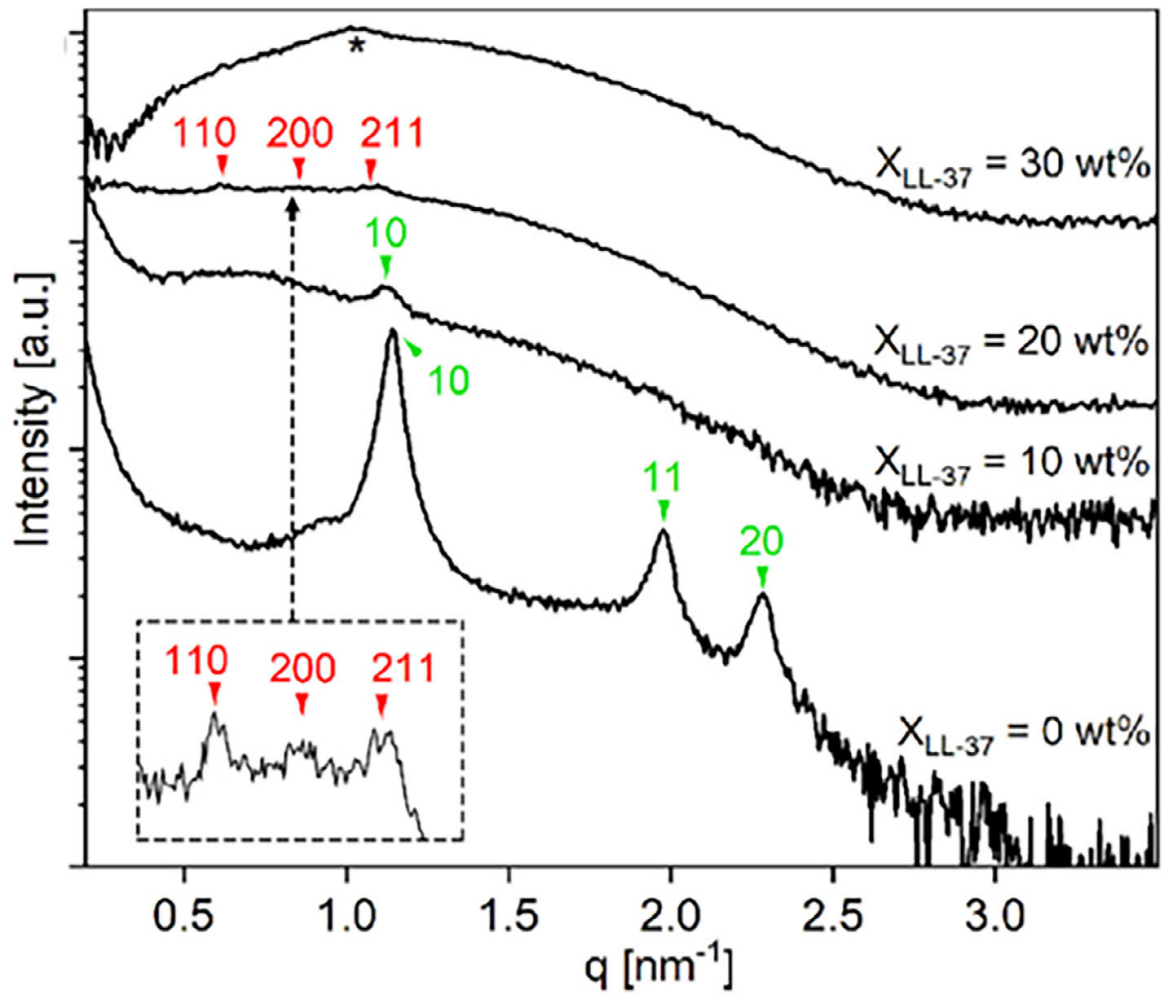
Small‐angle X‐ray scattering (SAXS) 1D scattering profile of nanocarriers comprised of oleic acid and glycerol monooleate with varying loadings of LL‐37. The ratio of oleic acid and glycerol monooleate was held at 3:7 and the pH of the samples was maintained at 6.5. Increasing concentrations of LL‐37 resulted in a transition from hexasomes, to inverse bicontinuous micelles to vesicles. Reproduced under the terms of the CC‐BY 4.0 license [158].

In drug delivery, antimicrobial peptides (AMPs) play a vital role in the innate immune response of all organisms and have recently found clinical applications due to their immune‐regulating, anti‐pathogenic, and anti‐cancer properties [158]. However, their poor stability in abnormal pH conditions is detrimental to their efficacy in therapeutics. For this reason, pH responsive nanocarriers have been developed to protect these AMPs from degradation. Gontsarik et al. have developed a detailed pH phase diagram of a nanocarrier comprised of oleic acid, glycerol monooleate and the AMP, human cathelicidin‐derived LL‐37, and were able to characterize the self‐assembled structure of the resulting carriers through SAXS (Figure 21) [159]. Colloidally stable nanocarriers of oleic acid and glycerol monooleate, maintained at a constant ratio (3:7) and pH (6.5), exhibited significant structural changes upon the addition of LL‐37. In the absence of LL‐37, the nanocarrier adopted a self‐assembled hexagonal structure, as evidenced by the characteristic peak ratio. Introduction of LL‐37 disrupts this hexagonal packing, resulting in an increase in the calculated lattice parameter from 6.4 nm to 6.5 nm. At a loading of 20 wt% of LL‐37, the nanocarrier adopts an inverse bicontinuous micelle configuration, as seen in its characteristic peak ratios. Further increases in LL‐37 loading results in vesicles, with a broad peak at 1.0 nm^−1^, which the authors attributed to weakly scattering bilayers in the vesicle.

To discern the role of LL‐37 loaded nanocarriers on Gram‐negative bacteria, a combination of peak and Porod analyses were carried out by Hong et al. [160]. AMP‐nanocarrier complexes have previously been found to be more effective against Gram‐negative bacteria, which was attributed to lipopolysaccharides (LPS) on the surface of these strains. Firstly, the authors examined the sole role of LL‐37 on LPS, where it was found that the AMP was incorporated into the LPS structure and formed an overall micellar structure. Further increases of LL‐37 resulted in a transition into a lamellar structure. Similarly, when examining the role of glycerol monooleate on the LPS structure, the overall complex resulted in cubosomes (essentially cubic lattices formed from micelles). When glycerol monooleate‐LL‐37 nanocarriers were combined with LPS, they adopted a lamellar structure that eventually transformed into an unstructured emulsion at high LPS concentrations. This indicates that the effectiveness of AMP‐loaded nanocarriers against Gram‐negative bacteria may stem from the LL‐37 release mechanism.

**FIGURE 22:**
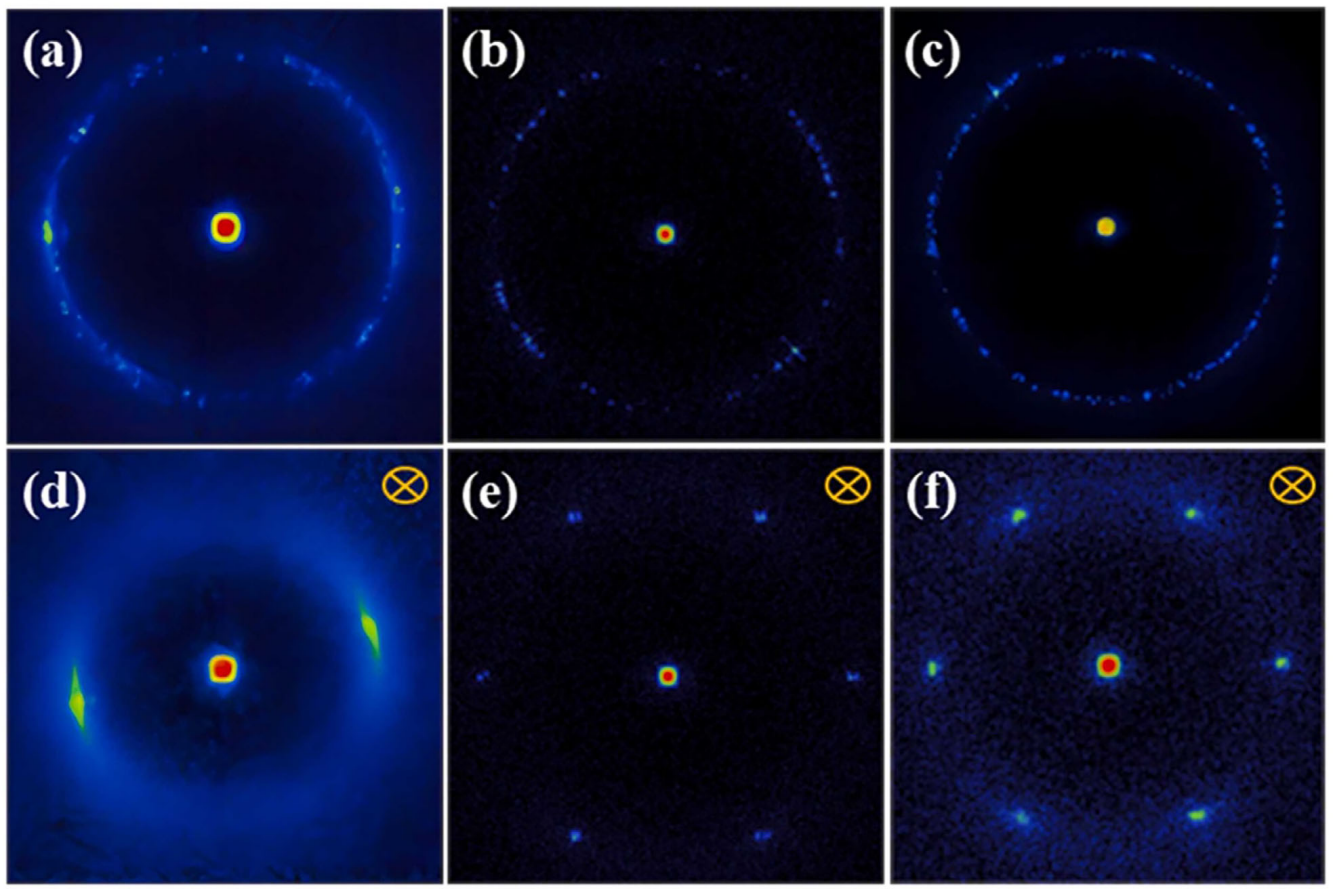
Before application of an electric field on mixtures of (a) brij56, (b) DTAB, (c) CTAB, and after application of the electric field on mixtures of (d) brij56, (e) DTAB, (f) CTAB. Reproduced with permission [161]. Copyright 2025, Elsevier.

Towards developing reinforced nanomembrane materials, a monomer mixture was dispersed in a matrix of hexagonal lyotropic liquid crystals (HLLCs) as to examine its effect on the structure, physical properties, and behaviors. Wang et al. performed a study where a monomer mixture of polyethylene glycol diacrylate and 2‐hydroxyethyl methacrylate was introduced into separate HLLC mixtures [161]. Generally, they observed that high monomer loadings resulted in phase coexistence of the hexagonal columnar phase with an isotropic liquid phase and monomer loadings were kept below this threshold as to ensure a homogenous hexagonal phase. Both mixtures, the binary surface/water and the ternary mixture of the monomer mixture/surfactant/water exhibited the peak ratio 1:√3:√4. Furthermore, upon application of an electric field, the long axis of the hexagonal cylinders aligned parallel to the field direction, which gave rise to a hexagonal 2D SAXS pattern. As can be seen, prior to application of the electric field (Figure 22a–c) the HLLCs are present in isotropic ordered domains (as evident in the continuous rings), however after the field is switched on, the HLLCs become reoriented and more anisotropic. Additionally, in the case of the CTAB and DTAB mixtures, the authors were able to extract an order parameter through azimuthal integration (in contrast to radial integration), followed by a Gaussian fitting. However, the same could not be achieved for the brij56 surfactant, owing to its strange behavior following the introduction of the electric field.

## 5 Grazing Incident SAXS (GISAXS)

**FIGURE 23:**
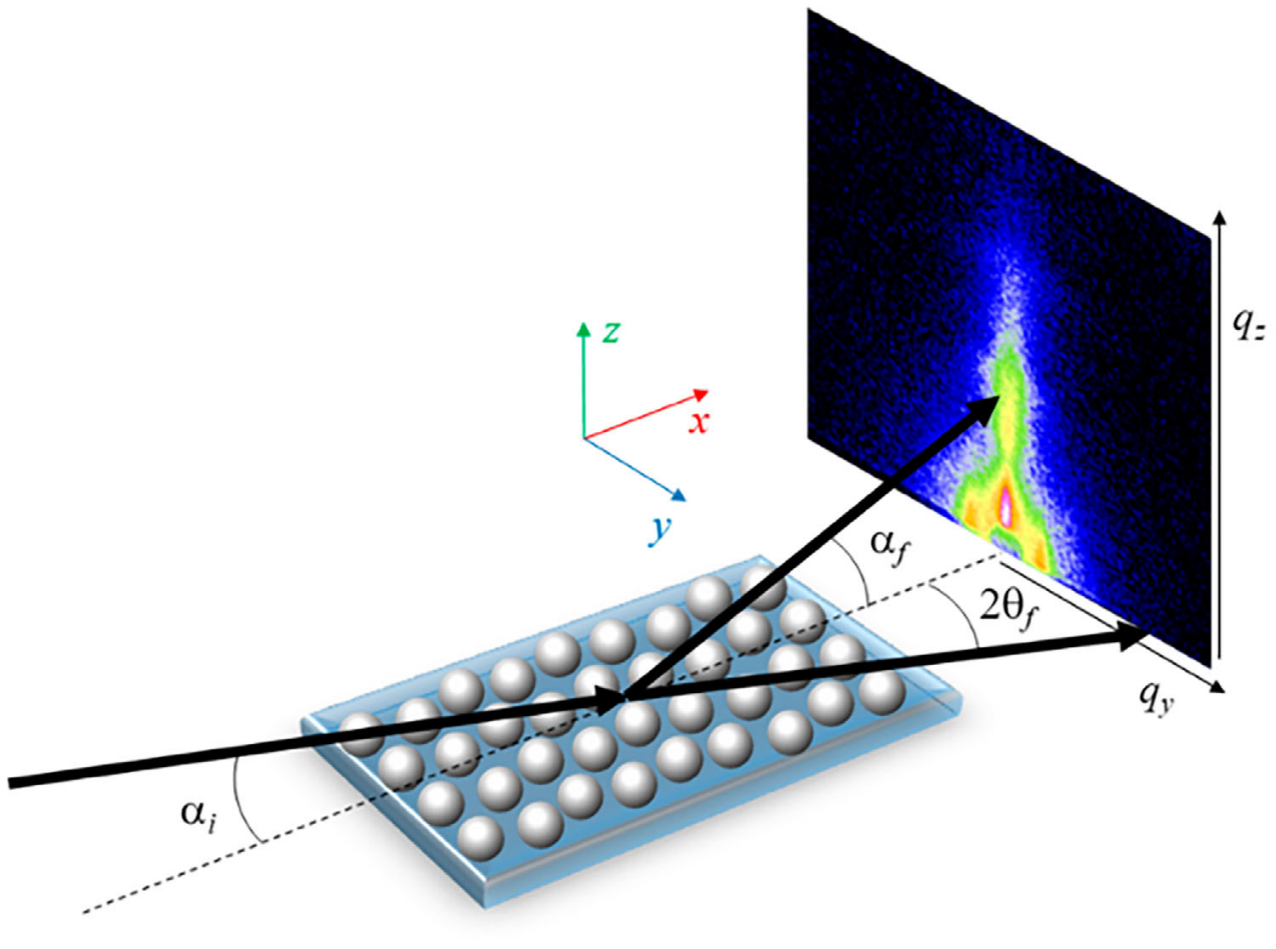
Schematic diagram of grazing incident small‐angle X‐ray scattering (GISAXS) setup. Typically, the instrument is set up such that the incident X‐ray beam is around the critical angle of the sample surface; the angle between the incident (α_i_) and scattered beams (α_f_) with the sample surface is typically <0.5°. In‐plane and out‐of‐plane scattering is obtained from a 2D detector, allowing for simultaneous collection of depth and surface morphologies. Reproduced under the terms of the CC‐BY 4.0 license [162].

All previously mentioned examples have been cases of transmission SAXS, where the X‐ray radiation is transmitted through the sample. Similarly, a useful characterization method can be implemented on thin films using a grazing incident SAXS (GISAXS) based technique, where the incident X‐ray beam is reflected from the surface, enabling characterization of nanoscale surfaces. GISAXS is largely similar to transmission SAXS, in which the X‐ray beam is incident on the sample surface at a small angle (<0.5°) [162]. This is known as the grazing incidence angle and is usually near the critical angle of the surface/substrate, such that total reflection is achieved (to minimize background scattering) [163]. This enables simultaneous characterization of nanoscale features which are perpendicular and parallel to the surface morphology (Figure 23). Furthermore, increasing the grazing angle above the critical angle allows for depth profiling of the sample and characterization of assemblies below the surface. In Figure 23, in‐plane scattering occurs along the *y*‐axis (*q*_y_) while out‐of‐plane scattering happens in the *z*‐axis (q_z_).

**FIGURE 24:**
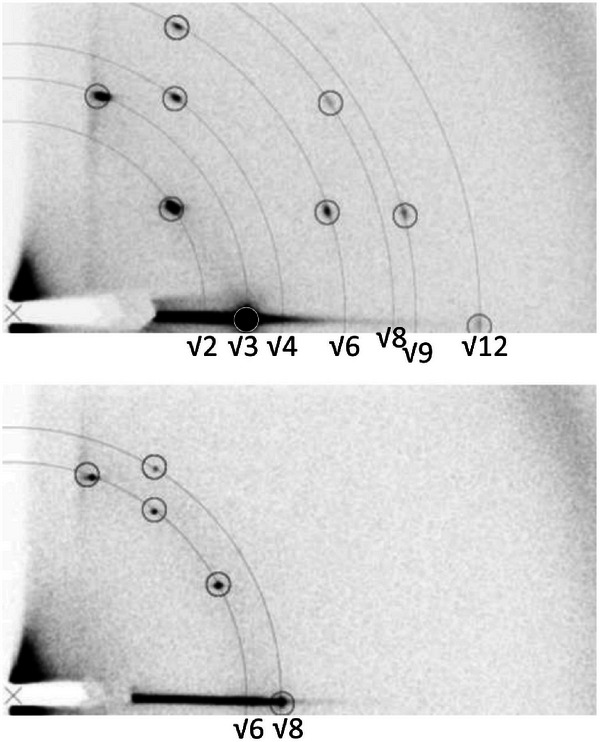
Grazing incident small‐angle X‐ray scattering (GISAXS) 2D pattern of a 1:4 (w/w) glycerol:phytantriol thin film, which was spin‐coated onto an SiO_2_ substrate. Detector images were taken at (top) 36% and (bottom) 90% relative humidity. The patterns and peak ratios correspond to the inverse bicontinuous cubic phases, diamond (*Pn* $\bar{3}$ *m*) and gyroid (*Ia* $\bar{3}$ *d*) diamond (*Pn* $\bar{3}$ *m*), respectively. Reproduced with permission [164]. Copyright 2014, American Chemical Society.

One example by Richardson et al. uses GISAXS to characterize prepared films of glycerol:phytantriol at a 1:4 (w/w) ratio [164]. A solution of this mixture was spin coated onto a silicon substrate to yield films with thicknesses ranging from 500 to 1500 nm. GISAXS was then performed with these films placed inside a humidity chamber, as to track the organizational transitions. 2D patterns were obtained at 36% and 90% relative humidity and are depicted in Figure 24. Herein, the bicontinuous inverse cubic phases are unidirectional and highly periodic, given that clearly distinct peaks are visible and up to seven orders of Bragg diffraction are observed. In a similar study, Rittman et al. also used GISAXS to track organizational changes in other lipid surfactants [165]. Here, phytantriol and two types of monoolein (research and industrial grade) were similarly prepared and subjected to 98% relative humidity. High‐quality 2D patterns were obtained from GISAXS, capturing the transition from gyroid (*la* $\bar{3}$ *d*) to diamond (*Pn* $\bar{3}$ *m*) in the case of phytantriol and the industrial grade monoolein, and an additional prior lamellar phase in the case of the academic grade monoolein.

**FIGURE 25:**
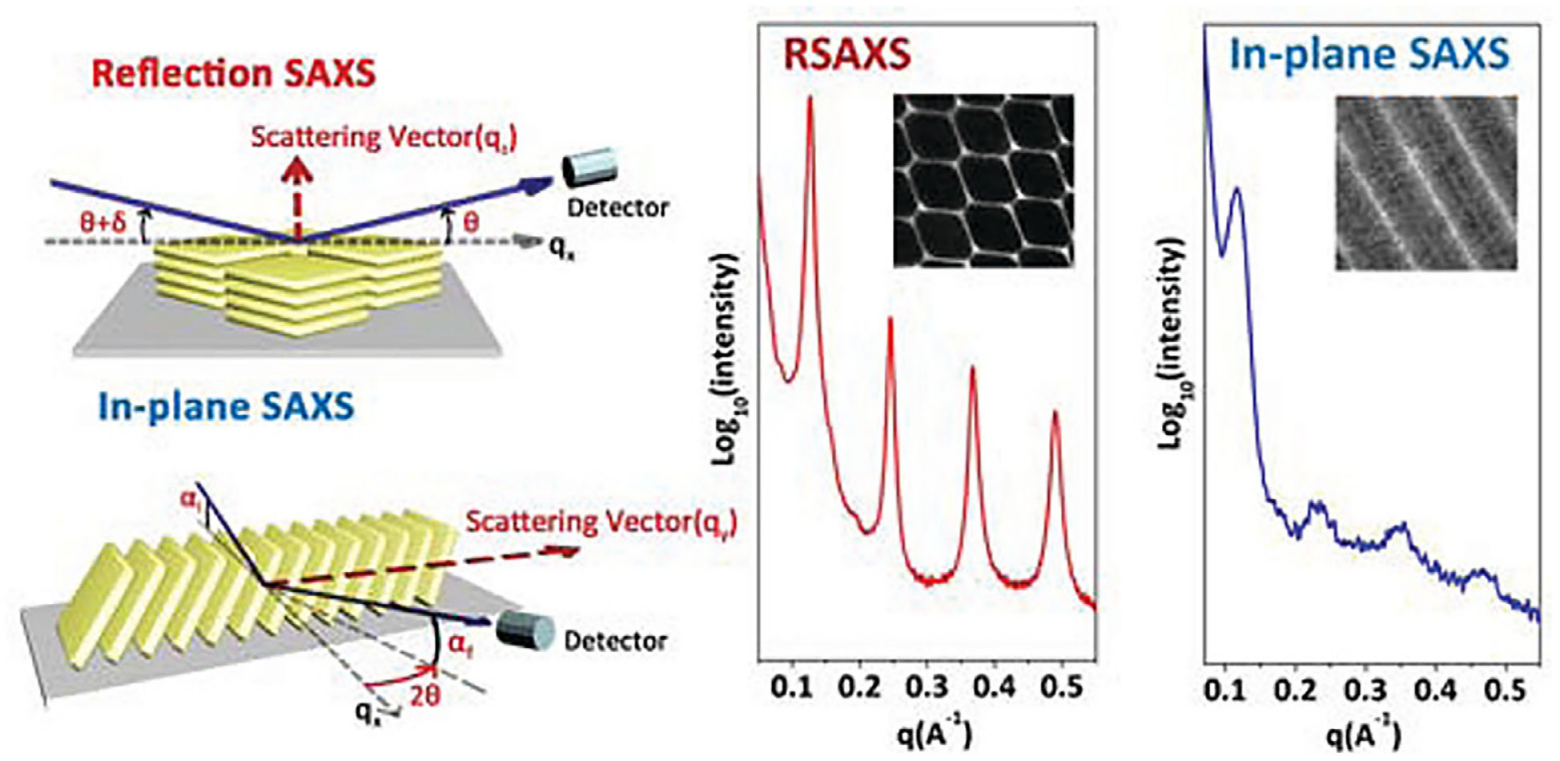
Differences in the collected scattering profiles of reflection small‐angle X‐ray scattering (RSAXS) and in‐plane SAXS. RSAXS produces strong Bragg reflections, owing to the scattering vector aligning with the detector. In contrast, in‐plane SAXS results in weaker reflections, as the scattering vector. Reproduced with permission [166]. Copyright 2021, American Chemical Society.

Similarly, Paik et al. successfully characterized superlattices of rhombic gadolinium trifluoride (GdF_3_) nanoplates using GISAXS (or reflection SAXS (RSAXS), as referred to in their study) [166]. It is important to note that although the instrument setup is similar to GISAXS, the authors appeared to have run RSAXS and in‐plane SAXS separately. Using the axis outlined in Figure 25, RSAXS and in‐plane SAXS represent the scattering wave vectors, *q*_z_ and *q*_y_, respectively. Self‐assembly was achieved through the liquid interfacial assembly by slow‐casting solutions of GdF_3_ nanoplates on top of solutions of various oligo‐ethylene glycols (Figure 25). Based on the TEM and SEM images, the GF_3_ nanoplates formed hexagonal columnar stacks growing parallel to their SiO_2_ substrate, with lamellar‐like stacking within the columns. While the authors didn't provide peak values (or ratios) for their 1D plots, the authors reported hexagonal columnar organization observed in RSAXS, while in‐plane SAXS revealed lamellar packing. From RSAXS, the primary peak occurs at 1.27 nm^−1^, corresponding to a center‐to‐center distance of 4.9 nm between the nanoplate columns. Subtraction of the nanoplate thickness (2.4 nm) from the center‐to‐center distance gives an intercolumn spacing of 2.5 nm. In contrast, in‐plane SAXS revealed weak Bragg reflections, corresponding to lamellar stacking of the nanoplates. Taken together, the results make for a compelling case for hexagonally packed columns comprised of stacks of GF_3_ nanoplates.

## 6 Metallomesogens and Liquid Crystal Complexes

The following section will explore metallomesogens and other molecules with distinctive geometries, aiming to highlight their unique and unconventional self‐assembled structures. Additionally, this section serves to highlight approaches toward characterization of the resulting supramolecular structures. Generally, this is largely accomplished through POM texture identification, followed by SWAXS pattern indexing/fitting [167, 168].

Metallomesogens are materials which contain a metal atom and exhibit LC properties [45, 169]. Through careful design of self‐assembling metal complexes, it becomes possible to potentially combine supramolecular structures with magnetic or luminescent properties [170, 171, 172]. Choice of the metal center allows for further customization of the electronic and physical properties. Furthermore, the coordinating ligands do not require inherent self‐assembly behavior, as supramolecular organization is possible through thoughtful metal complex design.

It is worth mentioning that in characterizing these self‐assembled structures composed of geometrically complex molecules, it is not always clear how the supramolecular organization is arranged. For this reason, Equation (19) is commonly used to determine the formula units occupied in a unit cell, which may then aid in the identification of the structure [173]. Here, a represents the calculated lattice parameter, *ρ* is the bulk density, *N*_A_ is Avagadro's number, and *M* is the molecular weight.

(19) $$Z=\frac{a^{3}\rho N_{A}}{M}$$

**FIGURE 26:**
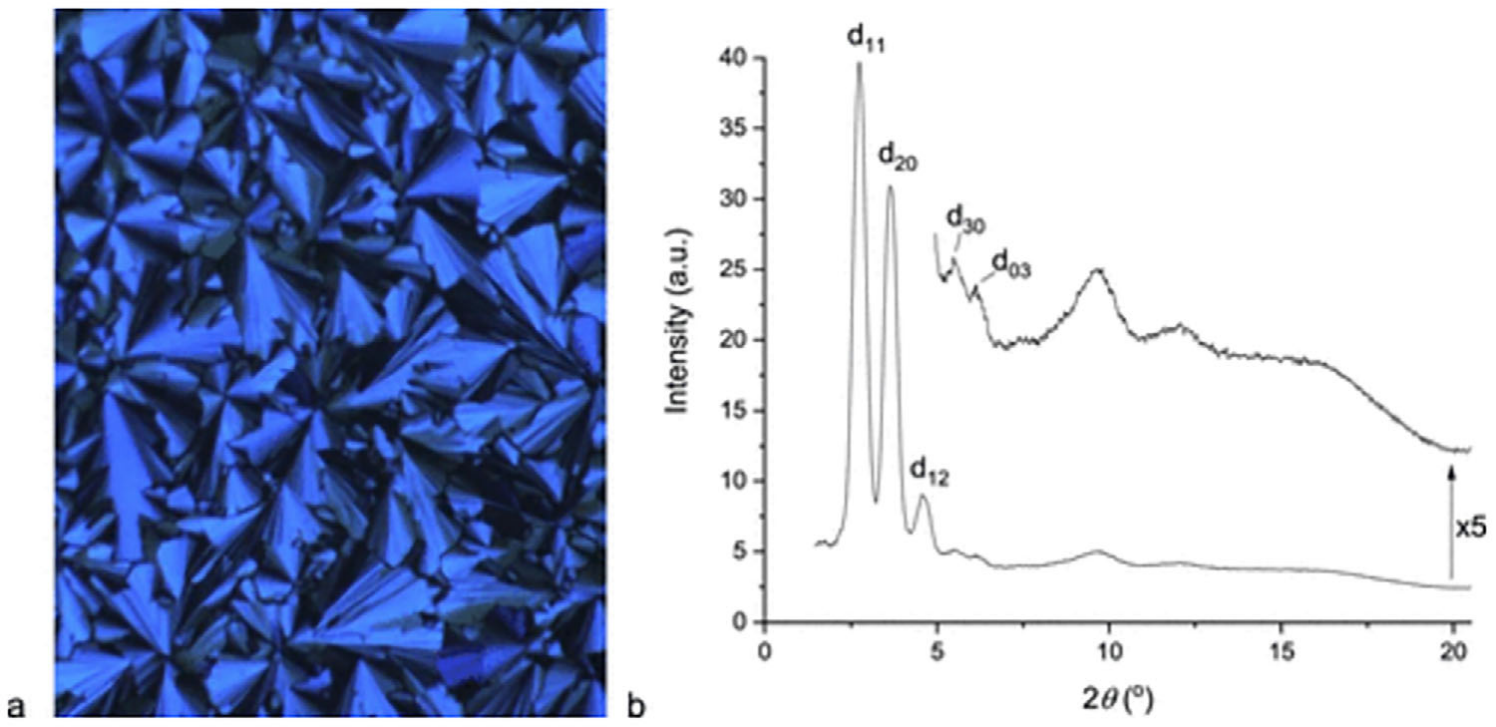
(a) Fan‐shaped polarized optical microscopy (POM) texture, which is commonly seen in columnar liquid crystal (LC) phases. (b) Small‐angle X‐ray scattering (SWAXS) profile of one of the rectangular columnar structures, which exhibit the Miller indices: (11), (20), (12), (30), (03). Reproduced under the terms of the CC‐BY 4.0 license [174].

For example, Parker et al. successfully synthesized a library of metallomesogens based on gold(III) complexes [174, 175, 176]. These complexes featured CNC pincer ligands and a monodentate acetylide ligand equipped with alkoxy arms of differing lengths. LC behavior was generated from the disk‐like structure of the gold(III) complexes, which enabled hexagonal columnar and rectangular columnar self‐assembled structures. POM initially revealed that several of the gold(III) complexes exhibited a birefringent texture corresponding to columnar LCs. SWAXS of the columnar samples revealed two distinct self‐assembled columnar organizations: hexagonal and rectangular. The hexagonal columnar structure was identified through the characteristic peak ratio, as previously mentioned. However, the observed reflections of the other structure were indexed to the following hk0 Miller indices: (11), (20), (12), (30), (03), which corresponds to a rectangular columnar structure (Figure 26). Furthermore, large, broad peaks were observed at 2*θ* = 10° and 12°, corresponding to a spacing of 0.95 and 0.72 nm, respectively, which the authors tentatively assigned to Au–Au correlations between complexes.

**FIGURE 27:**
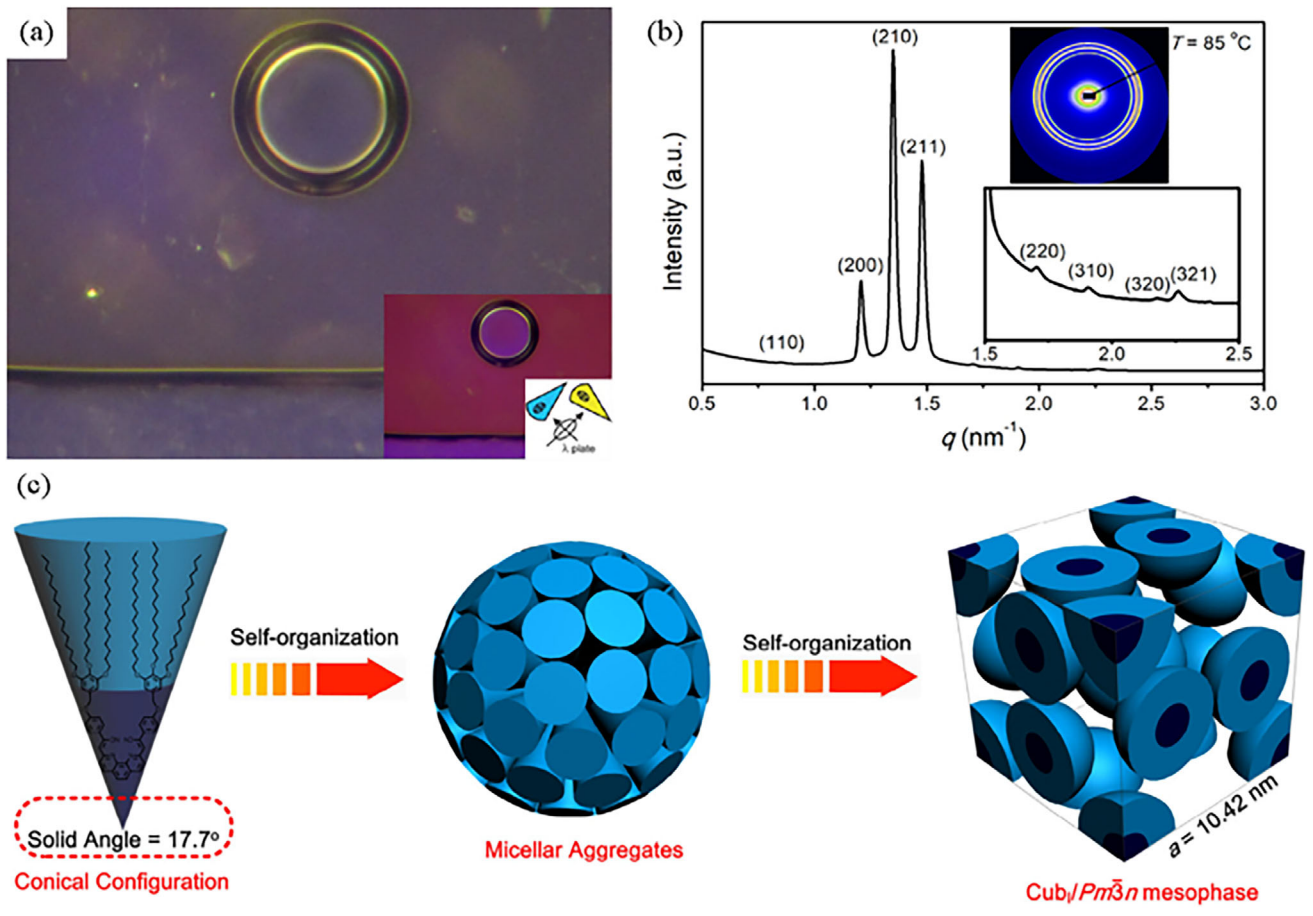
(a) Polarized optical microscopy (POM) texture of Phe/16, revealing non‐birefringent behavior. (b) Small‐angle X‐ray scattering (SWAXS) profile of Phe/16, which can be indexed to the cubic unit cell, *Pm*‐3*n*. (c) Proposed self‐assembly schematic, in order of increasing organization, from left to right. The V‐shaped Phe/16 molecule adopts a conical geometry, which forms spherical aggregates which it self‐assemble into the corresponding cubic structure. Reproduced with permission [177]. Copyright 2021, Elsevier.

Similarly, a small library of V‐shaped phenanthroline‐based hexacatenar molecules (abbreviated as Phe/N) were synthesized by Liu et al. and observed for its LC behavior [177]. Only one of the three synthesized molecules exhibited self‐assembly behavior, Phe/16. POM revealed that the sample did not exhibit birefringence, suggesting that the self‐assembled structure is likely cubic (due to equivalent optical axes). Furthermore, the sample was highly organized as SWAXS reveals several peaks, which were indexed as (110), (200), (210), (211), (220), (310), (320), and (321) (Figure 27). As a result, the structure was identified as a thermotropic micellar cubic phase, *Pm* $\bar{3}$ *n*. Using Equation (9), the authors deduced that this cubic phase houses eight spheres and the authors were able to determine that each sphere was composed of an aggregate of 41 Phe/16 molecules.

**FIGURE 28:**
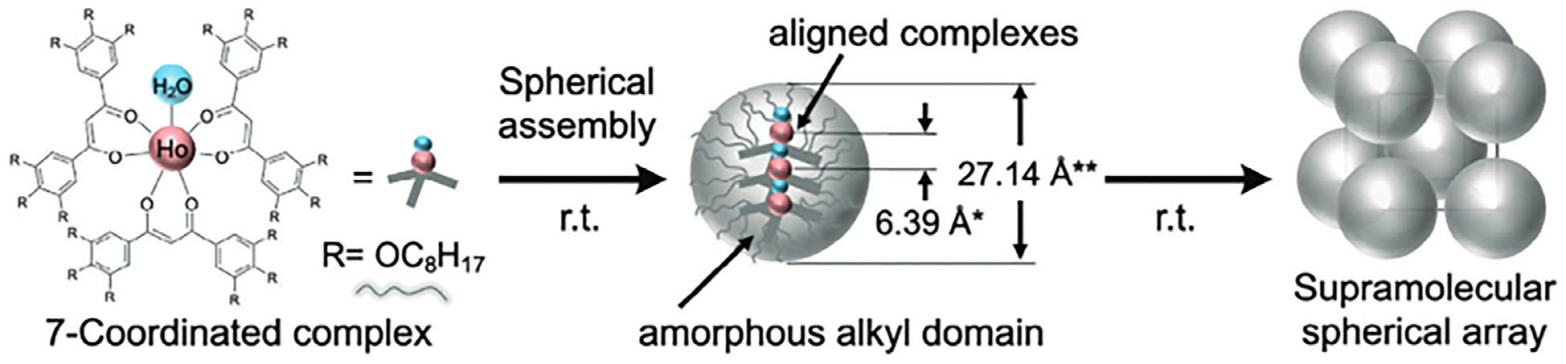
Self‐assembly schematic for the 7‐coordinate holmium complex, as determined by molecular dynamics simulations. Three HoC8 complexes form one supramolecular sphere, which in turn self‐assembles into the *Im* $\bar{3}$ *m* structure. Reproduced under the terms of the CC‐BY 4.0 license [178].

7‐coordinate lanthanide‐based metallomesogens were successfully synthesized by Komiyama et al. [178]. While 7‐coordinate complexes were accomplished with both holmium and europium, only the supramolecular organization of the holmium complex was discussed (HoC8, in which C8 is OC_8_H_17_ functionality). POM of this sample revealed non‐birefringent behavior, once again signalling cubic self‐assembly. The supramolecular structure exhibited several well‐resolved peaks using SWAXS: (110), (200), (211), (220), (310), (222), (321), (400), and (330/411). The observed reflections were then fitted to obtain a cubic structure, $Im\bar{3}m$. Afterwards, using Equation (10) and molecular dynamics simulations, the authors determined that the *Im* $\bar{3}$ *m* phase was micellar (rather than bicontinuous), with each sphere in the unit cell comprised of three HoC8 complexes. In these spherical aggregates, the Holmium core is surrounded by amorphous alkyl chains, which in turn enables supramolecular organization of the $Im\bar{3}m$ phase (Figure 28).

## 7 Conclusion

In summary, this review has highlighted the pivotal role of SAXS in the investigation of various self‐assembled materials. The instruments’ versatility, precision and compatibility with diverse sample types have made it an invaluable tool in LC characterization. Currently, active research in LC materials is highly interdisciplinary [47], with applications in addressing core challenges in their respective fields. For example, LC elastomers has seen great potential as bio‐inspired, soft robotics [179, 180]. Similarly, the stimuli responsive nature of LC materials has been harnessed to achieve programmable active materials [181]. Additionally, LCs have remained essential in the field of biomedical engineering owing to their potential as biochemical sensors/diagnostics [182], drug delivery carriers [182, 183, 184], as scaffolds toward tissue engineering [185, 186]. While most LC research is currently application‐focused, fundamental research on LCs remain essential. The field is advancing with the exploration of novel phases such as Frank–Kasper phases and liquid quasicrystals [187, 188, 189, 190], with the recent discovery of ferroelectric and antiferroelectric representing a frontier in the field of LCs [191, 192]. The development of these new LC technologies, as well as the advancement in our fundamental understanding of LC materials is dependent on methods which can resolve their complex nanostructures, rendering SWAXS essential in this regard. Advancements in synchrotron and lab‐based SWAXS capabilities are now resolving nanoscale features with unprecedented clarity. Techniques such as time‐resolved and in‐situ monitoring are particularly valuable, enabling a live, direct view into LC structural behavior under external stimuli.

SWAXS not only provides insight into various nanoscale features, phase transitions and structural dimensions, but has proved to be essential to researchers in establishing and understanding structure‐property relationships. Our discussion has highlighted creative uses of SAXS toward characterizing various types of LCs, showcasing its ability to drive advancements in materials chemistry. However, the largest limitation of SAXS is its accessibility, underutilization and challenges in data interpretation, but it is with hope that this review provides guidance by offering a comprehensive overview of SAXS fundamentals as well as applications and inspiration for further exploration. By leveraging the strengths of SAXS and addressing its limitations, future researchers have the opportunity to deepen our understanding of LC materials and unlock new possibilities for technological innovations.

## Conflicts of Interest

The authors declare no conflict of interest.

## Data Availability

The authors have nothing to report.
